# Comprehensive Characterization of a Novel E3-Related Gene Signature With Implications in Prognosis and Immunotherapy of Low-Grade Gliomas

**DOI:** 10.3389/fgene.2022.905047

**Published:** 2022-06-27

**Authors:** Shichuan Tan, Ryan Spear, Juan Zhao, Xiulian Sun, Pin Wang

**Affiliations:** ^1^ NHC Key Laboratory of Otorhinolaryngology, Qilu Hospital of Shandong University, Jinan, China; ^2^ Department of Neurology, Qilu Hospital of Shandong University, Jinan, China; ^3^ Brain Research Institute, Qilu Hospital of Shandong University, Jinan, China; ^4^ Department of Medicine, Rush University Medical Center, Chicago, IL, United States; ^5^ Department of Otorhinolaryngology, Qilu Hospital of Shandong University, Jinan, China; ^6^ The Key Laboratory of Cardiovascular Remodeling and Function Research, Chinese Ministry of Education, Chinese National Health Commission, Qilu Hospital of Shandong University, Jinan, China

**Keywords:** E3-related genes, prognosis, tumor immune microenvironment, risk signature, low-grade gliomas

## Abstract

Gliomas, a type of primary brain tumor, have emerged as a threat to global mortality due to their high heterogeneity and mortality. A low-grade glioma (LGG), although less aggressive compared with glioblastoma, still exhibits high recurrence and malignant progression. Ubiquitination is one of the most important posttranslational modifications that contribute to carcinogenesis and cancer recurrence. E3-related genes (E3RGs) play essential roles in the process of ubiquitination. Yet, the biological function and clinical significance of E3RGs in LGGs need further exploration. In this study, differentially expressed genes (DEGs) were screened by three differential expression analyses of LGG samples from The Cancer Genome Atlas (TCGA) database. DEGs with prognostic significance were selected by the univariate Cox regression analysis and log-rank statistical test. The LASSO-COX method was performed to identify an E3-related prognostic signature consisting of seven genes AURKA, PCGF2, MAP3K1, TRIM34, PRKN, TLE3, and TRIM17. The Chinese Glioma Genome Atlas (CGGA) dataset was used as the validation cohort. Kaplan–Meier survival analysis showed that LGG patients in the low-risk group had significantly higher overall survival time than those in the high-risk group in both TCGA and CGGA cohorts. Furthermore, multivariate Cox regression analysis revealed that the E3RG signature could be used as an independent prognostic factor. A nomogram based on the E3RG signature was then established and provided the prediction of the 1-, 3-, and 5-year survival probability of patients with LGGs. Moreover, DEGs were analyzed based on the risk signature, on which function analyses were performed. GO and KEGG analyses uncovered gene enrichment in extracellular matrix–related functions and immune-related biological processes in the high-risk group. GSEA revealed high enrichment in pathways that promote tumorigenesis and progression in the high-risk group. Furthermore, ESTIMATE algorithm analysis showed a significant difference in immune and stroma activity between high- and low-risk groups. Positive correlations between the risk signature and the tumor microenvironment immune cell infiltration and immune checkpoint molecules were also observed, implying that patients with the high-risk score may have better responses to immunotherapy. Overall, our findings might provide potential diagnostic and prognostic markers for LGG patients and offer meaningful insight for individualized treatment.

## Introduction

Glioma is a primary type of tumor that occurs in the brain and spinal cord. The World Health Organization (WHO) classification system categorizes gliomas from grade I (lowest grade) through grade IV (highest grade) according to the malignant histological features (Wesseling and Capper, 2018). Low-grade gliomas (LGGs), which are less aggressive than glioblastoma multiforme (GBM), mainly originate from astrocytes and oligodendrocytes. Patients with LGGs are categorized as WHO grade II–III gliomas. The standard management of patients with LGG primarily involves surgical resection followed by adjuvant radiotherapy and chemotherapy (Lombardi et al., 2020). Even after patients receive these standard clinical interventions, the highly invasive nature and heterogeneity of LGGs can still lead to high rates of tumor recurrence and noteworthy malignant progression (Brat et al., 2015; Xia et al., 2018; Gittleman et al., 2020). Furthermore, the prognosis of LGGs varies diversely due to tumor heterogeneity. LGG patients (mean age 41 years) are proposed to have survival averaging approximately 7 years, which is a significant sign of poor prognosis (Claus et al., 2015). Therefore, it is imperative to gain a more comprehensive understanding of the pathogenesis of LGG, identify effective and reliable biomarkers that could predict clinical outcomes, and formulate optimum therapeutic strategies.

Posttranslational modifications (PTMs) refer to covalent processing events of proteins which occur after synthesis and are normally mediated by diverse enzymes. Ubiquitination is a crucial posttranslational modification of a protein. It is an ATP-dependent reversible process mediated by the ubiquitin-proteasome system (UPS), including E1 ubiquitin–activating enzymes, E2 ubiquitin–conjugating enzymes, E3 ubiquitin-protein ligases, and deubiquitinating enzymes (DUBs) (Reyes-Turcu et al., 2009; Schulman and Harper, 2009; Buetow and Huang, 2016; Stewart et al., 2016). The dysregulation of UPS is largely involved in numerous biological functions, including cell cycle progression, cell proliferation, apoptosis, gene transcription, metastasis, transcriptional regulation, signaling, and inflammation (Deng et al., 2020). Accordingly, abnormal ubiquitination may contribute to various human pathologies such as neurodegeneration (Stieren et al., 2011; Popovic et al., 2014), autoimmune responses (Zangiabadi and Abdul-Sater, 2022), and oncogenic processes (Rape, 2018). In the UPS, E3 ubiquitin ligase serves as the essential part of the ubiquitination process owing to its substrate specificity (Zheng and Shabek, 2017). UPS is stringently regulated by E3 ligases that convey the specificity of ubiquitination. In particular, ubiquitin molecules are transferred from ubiquitin-conjugating enzymes to specific substrates by E3 ubiquitin–protein ligases. The misregulation of UPS led by mutations in E3-related genes (E3RGs) is highly correlated with poor prognosis of cancers (Seeler and Dejean, 2017). Accumulating studies have demonstrated the tremendous contribution of E3-related proteins in glioma pathogenesis. For instance, MYH9-mediated ubiquitination of GSK-3β promotes malignant progression and chemoresistance in glioma (Que et al., 2022). The degradation of TUSC2 induced by NEDD4 facilitates glioblastoma progression (Rimkus et al., 2022). PARK2, frequently mutated in glioma, acts as a tumor suppressor by boosting ubiquitination-dependent degradation of β-catenin, which results in attenuation of Wnt signaling (Veeriah et al., 2010; Lin et al., 2015). Tripartite motif-containing protein 11 (TRIM11), overexpressed in glioma, promotes proliferation, invasion, migration, and glial tumor growth *via* the induction of EGFR (Di et al., 2013). In addition, many E3 ligases have been reported to play vital roles in glioma carcinogenesis *via* the regulating PI3K/Akt pathway, such as SCF^β−TrCP^ (Warfel et al., 2011), TRAF6 (Feng et al., 2014), and TRIM21 (Lee et al., 2017). Based on the significant function of E3 ligases in cancer pathogenesis, therapeutics targeting UPS have shown promising effects in clinical trials against cancers, such as PROTAC (proteolysis-targeting chimeric) (Qi et al., 2021). Two PROTAC strategies targeting CDK4 and/or CDK6 have been tested in glioma cells and are expected in clinical trials soon (Zhao and Burgess, 2019). Given the crucial roles of E3-related genes in glioma, the mechanism underlying the relationship between E3-related genes and the prognosis of LGG needs to be further addressed.

In the present study, a comprehensive analysis of E3RGs in LGG was conducted. Transcriptome data and clinical data of LGG samples were downloaded from The Cancer Genome Atlas (TCGA) database. Differentially expressed genes (DEGs) with prognostic significance were screened and identified. Subsequently, an E3-related prognostic signature was constructed, and the nomogram based on the risk signature was established to predict the prognosis of LGG. Meanwhile, enrichment analyses of risk-related differentially expressed genes and substrates of the risk signature genes were performed to disclose the underlying mechanism of LGG progression. Finally, the correlation between the risk signature and tumor immunity was analyzed. Our work provided an effective clinical tool for LGG prognosis prediction and preliminarily explored the biological functions and immune processes involved in the signature and the relative regulatory networks.

## Materials and Methods

### Datasets and E3-Related Gene Acquisition

The transcriptome sequencing data and corresponding clinical data of primary LGG were obtained from the TCGA database (https://portal.gdc.cancer.gov/) and the Chinese Glioma Genome Atlas (CGGA) database, respectively, (http://www.cgga.org.cn/). The TCGA LGG cohort was selected as the training set, which included 451 tumor samples and five normal brain samples. The CGGA cohort (DataSet ID: mRNAseq_693) was chosen as the validation set, containing 240 primary LGG patients. The samples with incomplete clinical information and overall survival < 30 days had been excluded. Count data from TCGA and FPKM data from CGGA were applied for further analysis. The GSE68848 and GSE4290 datasets procured from the Gene Expression Omnibus database (GEO; https://www.ncbi.nlm.nih.gov/geo/) were utilized to validate the expression of the signature genes. The 630 E3RGs utilized in this study were collected from the ESBL database (https://esbl.nhlbi.nih.gov/Databases/KSBP2/Targets/Lists/E3-ligases/) and iUUCD2.0 database (http://iuucd.biocuckoo.org/) (Supplementary Table S1).

### Identification of Differentially Expressed E3-Related Genes

E3-related DEGs between LGG tissues and normal brain tissues were analyzed using the “limma,” “edgeR,” and “DESeq2” R packages with the cut-off criteria of |log_2_FC| ≥ 1 and *p* < 0.05 (Robinson et al., 2010; Love et al., 2014; Ritchie et al., 2015). The raw count data of the TCGA LGG cohort were used as the input for limma, edgeR, and DESeq. Volcano plots were generated to display DEG distribution from three algorithms mentioned earlier with the “tinyarray” R package. Venn diagrams were intersected to obtain the overlapping enriched terms also with the “tinyarray” R package.

### Identification of E3-Related Differentially Expressed Genes With the Prognostic Value

The CPM of genes and clinical information were used for the subsequent analyses. The univariate Cox regression analysis and log-rank statistical test with the cut-off criteria of *p* < 0.05 for E3-related DEGs were applied to select the genes with prognostic significance, using “survival” and “survminer” packages in R software. Venn diagrams were used to present the intersection of the enriched genes from Cox regression analysis and log-rank analysis.

### Construction and Validation of the Prognostic Signature

To minimize the overfitting high-dimensional prognostic E3-related DEGs, least absolute shrinkage and selection operator (LASSO) regression analysis was performed with the “glmnet” R package (Friedman et al., 2010). Multivariate Cox regression analysis was conducted to construct prognostic models with the R package “My.stepwise”. Hazard ratios (HRs) and 95% confidence intervals (CIs) were reported where applicable. The median risk score was calculated to categorize the LGG patients into high-risk and low-risk groups, based on the following formula:

$\text{Risk}\;\text{score}=\overset{n}{\underset{i=1}{\sum }}\text{Coe}{\text{f}}_{\text{i}}\times \text{Exp}{\text{r}}_{\text{i}},$

(where $\text{Coe}f_{i}$ is the coefficient and $\text{Exp}r_{i}$ is the expression level of each intersected gene).

A Kaplan–Meier survival curve was used to determine the differences in overall survival using the R package “survival”. Time-dependent receiver operating characteristic (ROC) analysis was executed to evaluate the prognostic accuracy of the seven-E3RG risk signature using R package “timeROC” (Blanche et al., 2013). The survival analysis result was presented by the R package “tinyarray” and “patchwork.”

### Development and Evaluation of the Nomogram

To assess whether the seven-E3RG prognostic risk signature can be utilized as an independent prognostic factor, univariate and multivariate Cox regression analyses were performed using R package “survival” and “My.stepwise.” The nomogram was constructed with independent prognostic parameters using the “rms” R package, and the calibration curves were utilized to reflect the accuracy of the nomogram.

### Risk-Related Differentially Expressed Gene Analysis

LGG patients were divided into high-risk and low-risk groups according to the median risk score. The raw count data of the TCGA LGG cohort were used as the input data for limma, edgeR, and DESeq. “limma,” “edgeR,” and “DESeq2” R packages with the cut-off criteria of |log_2_FC| ≥ 1 and *p* < 0.05 were applied to two groups for DEG screening. Volcano plots were generated to display DEG distribution from three algorithms. Venn diagrams were intersected to obtain the overlapping enriched terms.

### Functional Enrichment and GSEA

Gene Ontology (GO) and Kyoto Encyclopedia of Genes and Genomes (KEGG) analyses were performed utilizing the “clusterProfiler” package to predict the biological function and related pathways (Yu et al., 2012). The top five enriched terms in the biological process (BP), cellular component (CC), and molecular function (MF) were visualized using “ggplot2” and “enrichplot” R packages. KEGG pathways with P. adjust<0.01 were chosen for presentation. Gene set enrichment analysis (GSEA) was conducted using GSEA v4.2.3 software with hallmark gene sets.

### Immune Microenvironment Analysis

The tumor immune microenvironment (TIME) cell infiltration characterization was evaluated by the single-sample gene set enrichment analysis (ssGSEA) with the “GSVA” R package (Hänzelmann et al., 2013). An ESTIMATE algorithm was used to evaluate the immune and stromal activity in the LGG microenvironment with the “estimate” R package (Yoshihara et al., 2013). The correlation analysis was performed based on Pearson’s correlation coefficient and presented using R packages “corrplot” and “circlize” (Gu et al., 2014).

### Protein–Protein Interaction Network Analysis

A PPI network was constructed based on the substrates of E3RG signature with required interaction score > 0.4 using the STRING database (https://cn.string-db.org/) and visualized using Cytoscape (version 3.9.1) (Shannon et al., 2003; Szklarczyk et al., 2019). The top 10 hub genes in the PPI network were identified using the MCC algorithm with the CytoHubba plugin in Cytoscape.

## Results

### Identification of Differentially Expressed E3-Related Genes With the Prognostic Value in Low-Grade Gliomas

**FIGURE 1:**
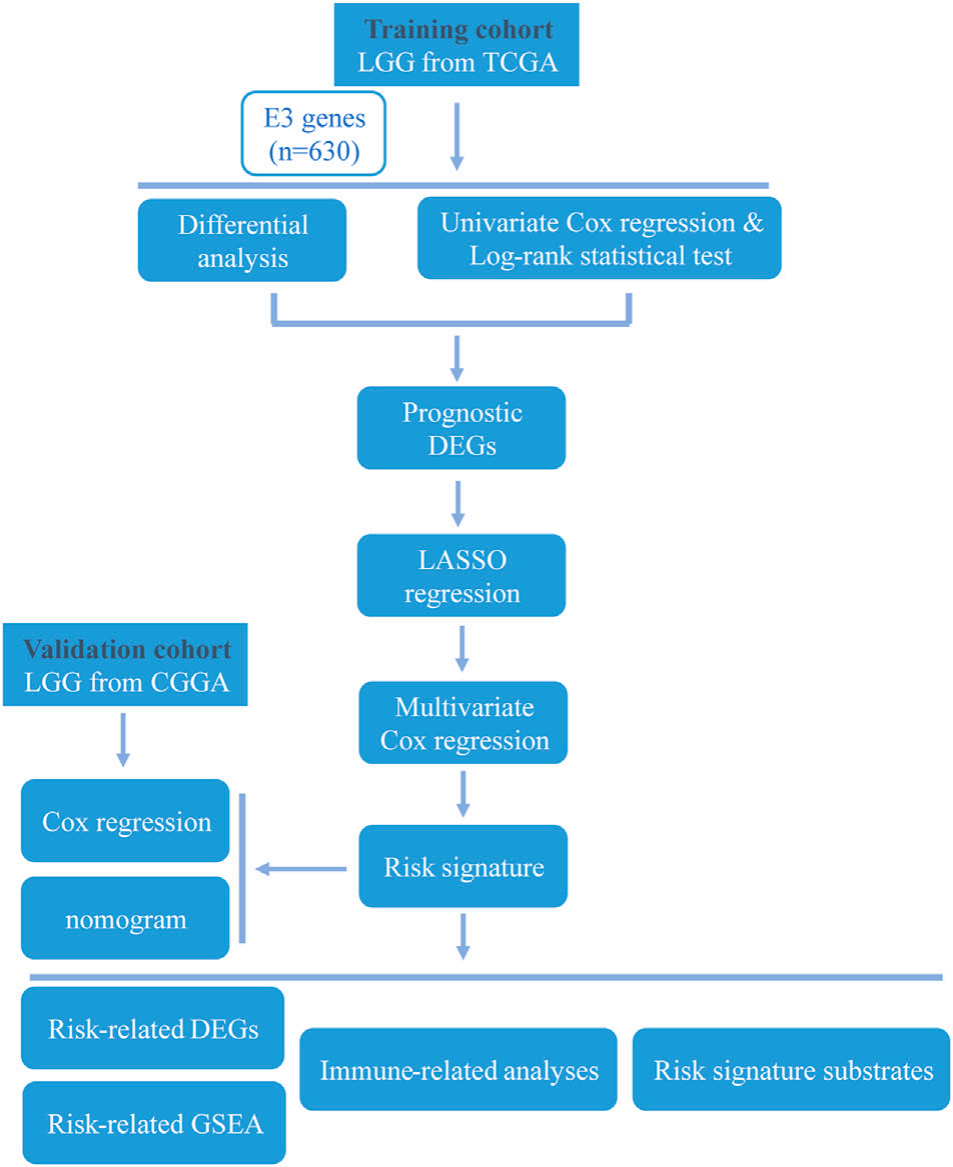
Workflow of the study.

**FIGURE 2:**
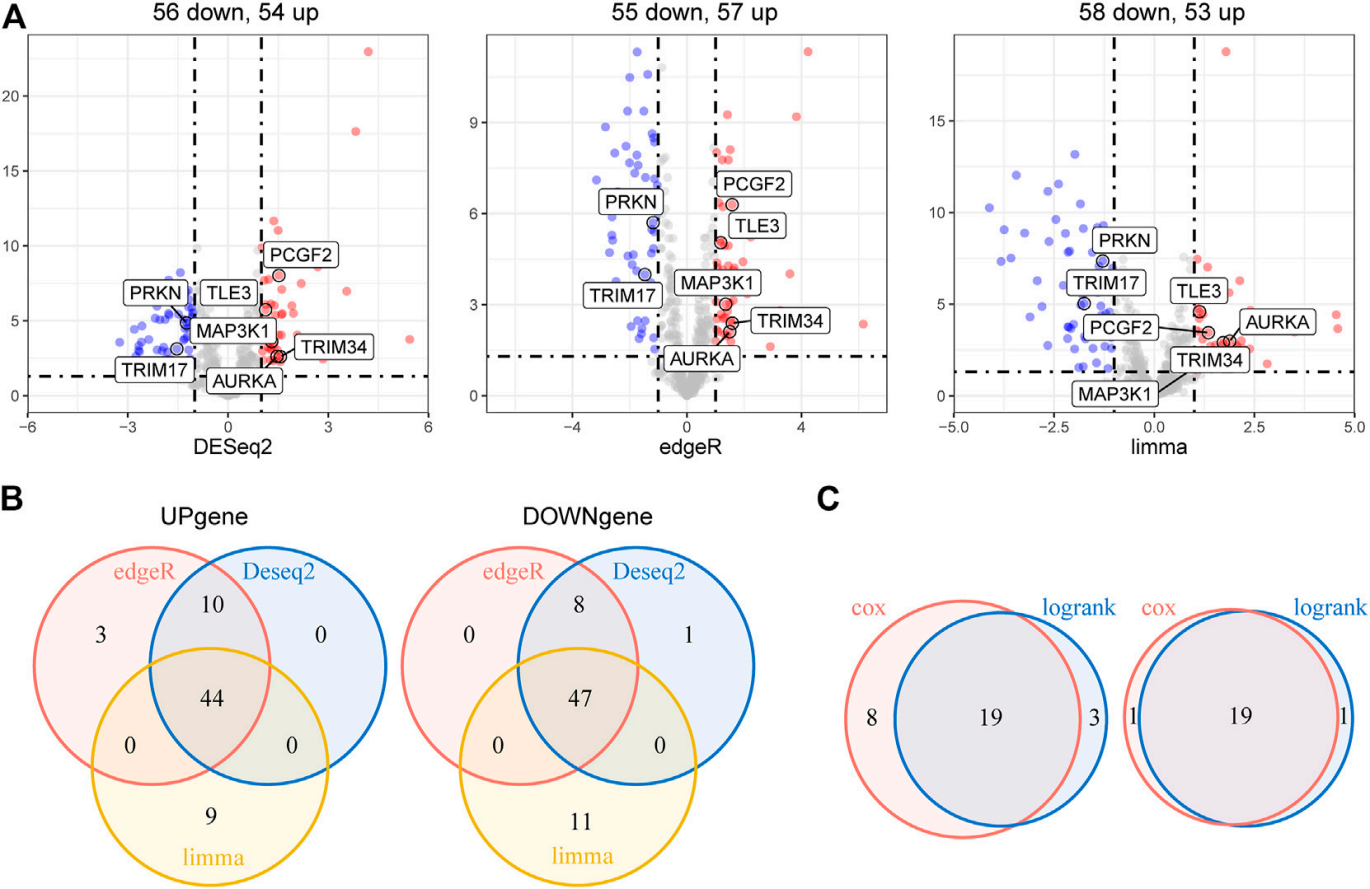
Identification of E3-related DEGs with prognostic value in LGG. **(A)** Volcano plot of E3-related DEGs between 451 LGG samples and five normal brain samples identified using edgeR, limma, and DESeq2 algorithms, with the cut-off criterion *p* < 0.05 and |log_2_FC| ≥ 1. Blue dots: significantly downregulated genes; red dots: significantly upregulated genes. **(B)** Venn diagram of the overlapping E3-related DEGs screened by the three differential expression analyses. **(C)** Venn diagram of the intersection for overall survival–correlated E3-related DEGs identified using univariate Cox regression analysis and log-rank statistical test with the cut-off criteria of *p* < 0.05. Left, predicted favorable prognosis; right, predicted poor prognosis.

The overall study workflow is presented in Figure 1. The TCGA cohort was used as the training set. The transcriptome and clinical data from TCGA included 451 tumor samples and five normal brain samples. The 630 E3RGs were selected and applied in this study (Supplementary Table S1). Three differential expression analyses were performed. According to the | log_2_ FC | > 1.0 and *p* < 0.05, DEGs were displayed in volcano plots **(** Figure 2A). The overlapping of DEGs from three differential expression analyses indicated that 44 genes were upregulated and 47 genes were downregulated in tumor samples (Figure 2B). Subsequently, DEGs were further analyzed by univariate Cox regression analysis and log-rank statistical test to evaluate the prognostic significance. In total, 38 E3RGs with a significant prognostic value were obtained by the intersection of results from both analyses (Figure 2C).

### Construction of E3-Related Gene Prognostic Risk Signature in Low-Grade Gliomas

**FIGURE 3:**
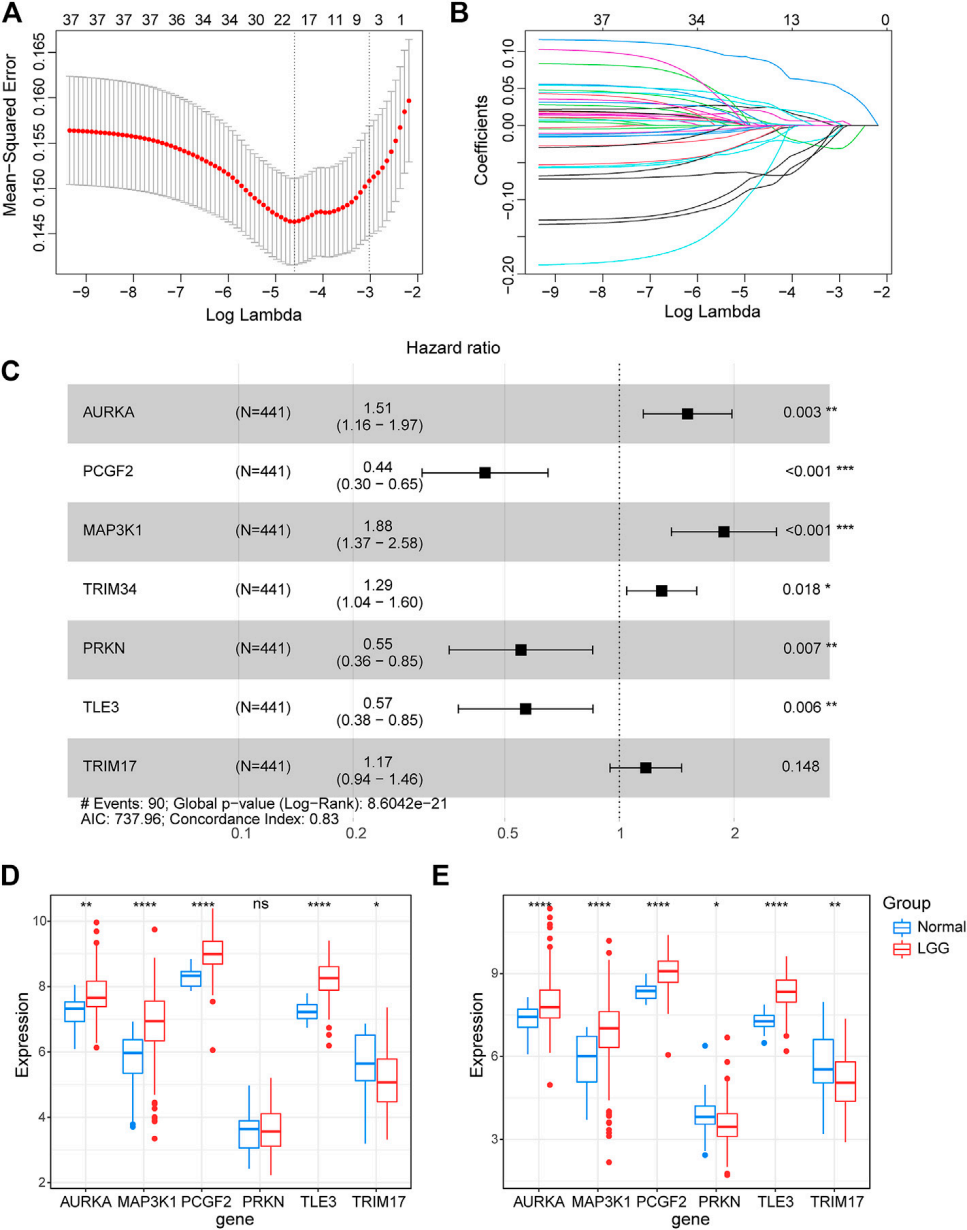
Construction of the prognostic E3-related signature. **(A)** LASSO regression cross-validation for tuning parameter lambda selection. **(B)** Coefficient profiles of the LASSO regression analysis. **(C)** Seven optimal prognostic E3-related DEGs selected by multivariate Cox regression analysis are shown in the forest plot. **(D)** Expression of the signature genes in the GSE4290 dataset. **(E)** Expression of the signature genes in the GSE68848 dataset. (*p < 0.05; **p < 0.01; ***p < 0.001; ****p < 0.0001; ns, not significant).

To further explore the prognostic value of E3RGs in LGGs, 38 overall survival–associated E3RGs were incorporated into the LASSO regression (Liu et al., 2021; 2022a; 2022b; 2022c), 20 of which were selected for further multivariate Cox regression analysis (Figures 3A,B). Following this, a seven-E3RG prognostic signature significantly correlated with LGG prognosis was developed by performing multivariate Cox regression analysis and shown in the forest plot (Figure 3C). The risk score was calculated for each LGG patient by the following formula:

Riskscore=(0.4121493)∗ AURKA+ (-0.8124184)∗PCGF2+ (0.6318466)∗MAP3K1+ (0.2558092)∗TRIM34+(-0.5951688)∗PRKN+(-0.5669701)∗TLE3+(0.1586579)∗TRIM17.

**FIGURE 4:**
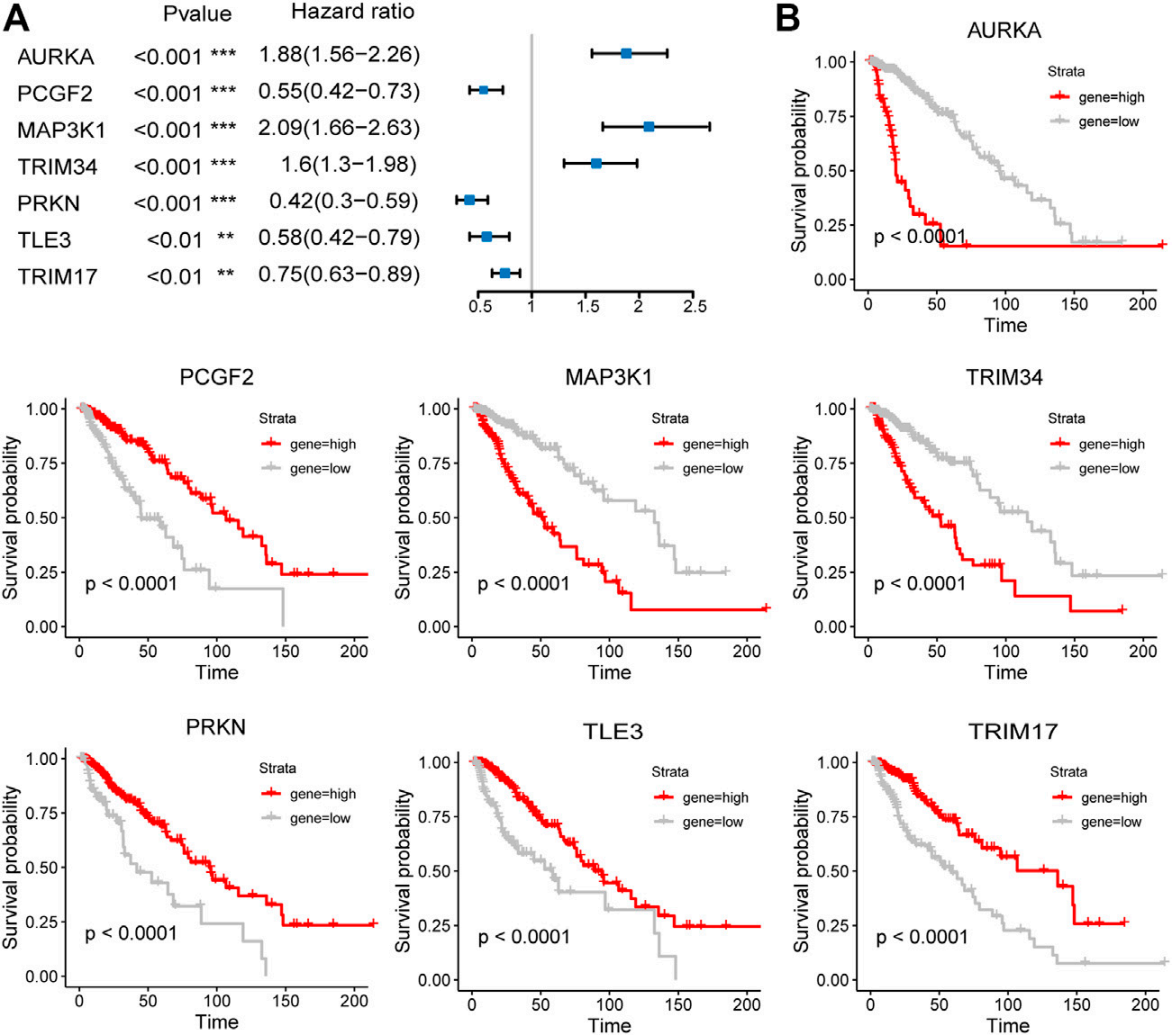
Prognostic analysis of the seven-E3RG risk signature. Univariate Cox regression analysis **(A)** and Kaplan–Meier curves **(B)** of the seven-E3RG prognostic signature (**p < 0.01; ***p < 0.001).

The expression of the signature genes was validated in both GSE4290 and GSE68848 datasets and proved consistent with that in the TCGA cohort (Figures 3D,E). Univariate Cox regression analysis revealed that seven signature genes were all strongly correlated with the prognosis (Figure 4A). As shown in the Kaplan–Meier curves, four genes of the E3RG signature were considered to have favorable prognostic effects, while three were considered to have poor prognostic effects (Figure 4B).

**FIGURE 5:**
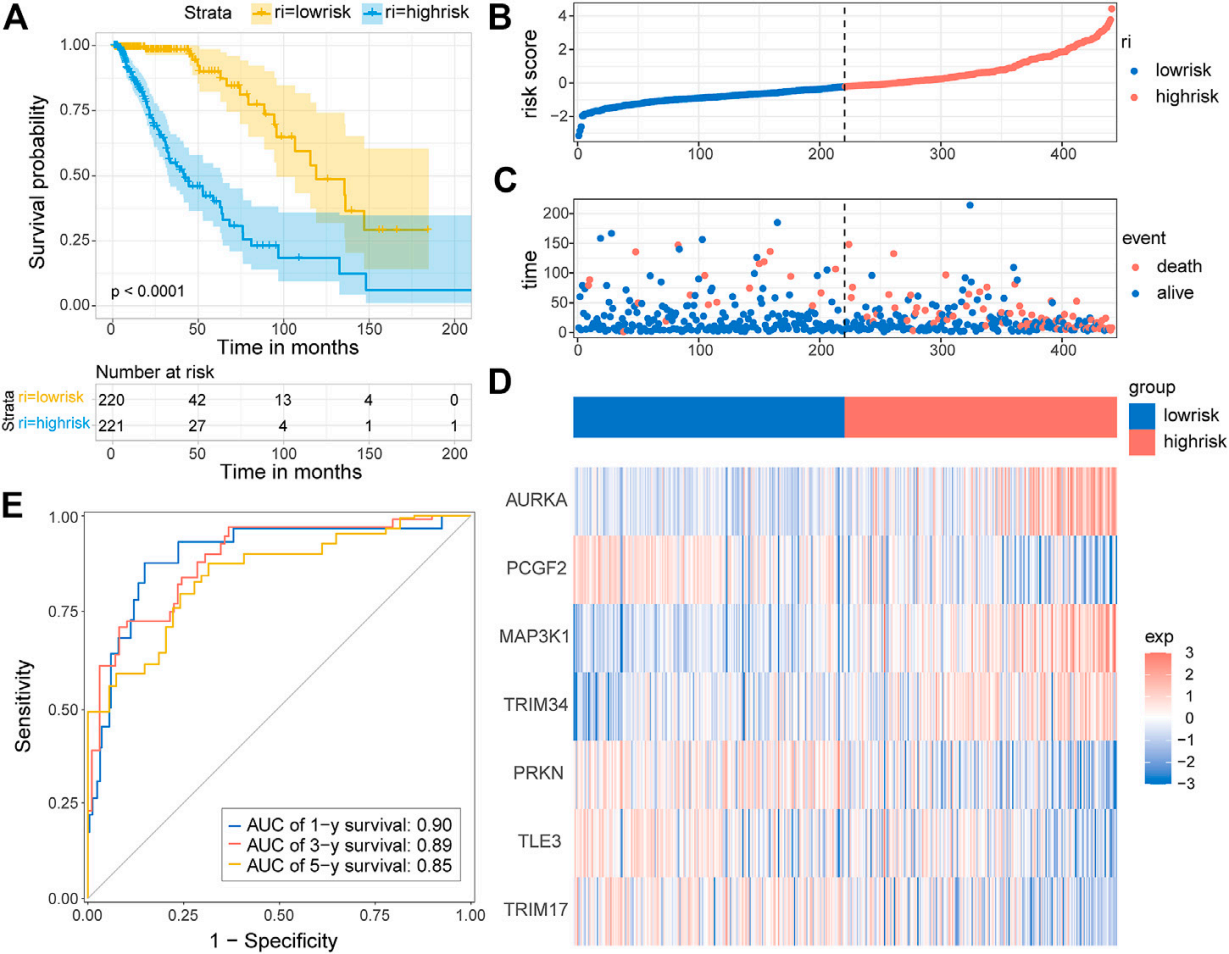
Distribution and prognostic analysis of the E3RG prognostic signature in the TCGA cohort. **(A)** Kaplan–Meier curves of overall survival for the patients in the high-risk group and the low-risk group of the TCGA cohort. The distribution plots **(B)**, corresponding survival status **(C)**, and the expression of the seven signature genes **(D)** in the TCGA cohort. **(E)** Time-dependent ROC curve validation of the predictive capacity of the risk signature in the TCGA cohort.

Based on the seven-E3RG risk signature, patients were divided into the high-risk and low-risk subgroups according to the median risk score in TCGA cohorts. The resulting Kaplan–Meier curve displayed a significant difference in overall survival between the LGG patients in the high-risk and the low-risk group, suggesting the established signature effectively predicts survival (Figure 5A; *p* < 0.0001). The overall survival of patients with low-risk scores was significantly higher than that of patients with high-risk scores. The genes referred to in the signature were remarkably differentially expressed in the high-risk group and the low-risk group. The expression of AURKA, MAP3K1, and TRIM34 was lower in low-risk patients than in high-risk patients, while PCGF2, PRKN, TLE3, and TRIM17 expressions were higher in patients with low-risk scores than in those with high-risk scores. The distribution of risk score, survival status, and the expression of the signature genes is shown in Figures 5B–D. To evaluate the predictive effect of the prognostic model, 1-year, 3-year, and 5-year time-dependent ROC curves were plotted, and the concordance index was calculated. The area under the curve (AUC) values were 0.9 (1-year ROC), 0.89 (3-year ROC), and 0.85 (5-year ROC) (Figure 5E). Given the results earlier, the risk signature presented a superior predictive capacity for LGGs.

### Validation of the Risk Signature in the Chinese Glioma Genome Atlas Cohort

**FIGURE 6:**
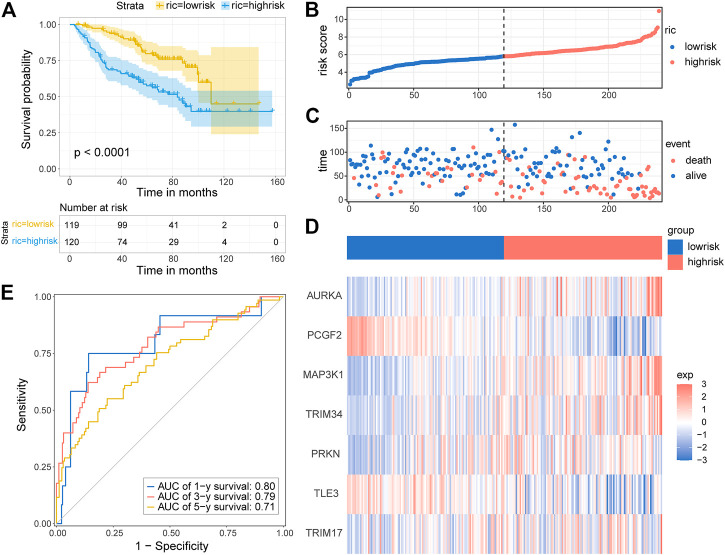
Validation of the E3RG prognostic signature in the CGGA cohort. **(A)** Kaplan–Meier curves of the overall survival in the high-risk group and low-risk group of the CGGA cohort. The distribution plots **(B)**, survival status **(C)**, and the expression of seven signature genes **(D)** in the CGGA cohort. **(E)** Time-dependent ROC curve validation of the predictive capacity of the risk signature in the CGGA cohort.

To test whether the prognostic gene signature has similar predictive performance and accuracy in other LGG cohorts, the CGGA cohort was used as a validation set. In the CGGA cohort, the patients were divided into low-risk and high-risk groups by the median risk score with the same formula calculated from the TCGA cohort (Figure 6B). Consistent with the results obtained from the training set, survival analysis using the Kaplan–Meier method exhibited a better prognosis for patients in the low-risk group (Figure 6A, *p* < 0.0001). The distribution of risk score and survival status is shown in Figures 6B,C. A heatmap of gene expression in the CGGA cohort is presented in Figure 6D, based on the risk score. The predicted AUCs of 1 year, 3 year, and 5 year are 0.80, 0.79, and 0.71, respectively (Figure 6E). Prognostic analyses showed similar results. These results demonstrated that the E3RG risk signature was positively correlated with LGG prognosis.

### Independent Prognostic Value of the E3-Related Gene Risk Signature in the Cancer Genome Atlas and the Chinese Glioma Genome Atlas Low-Grade Glioma Cohorts and Construction of a Nomogram

**FIGURE 7:**
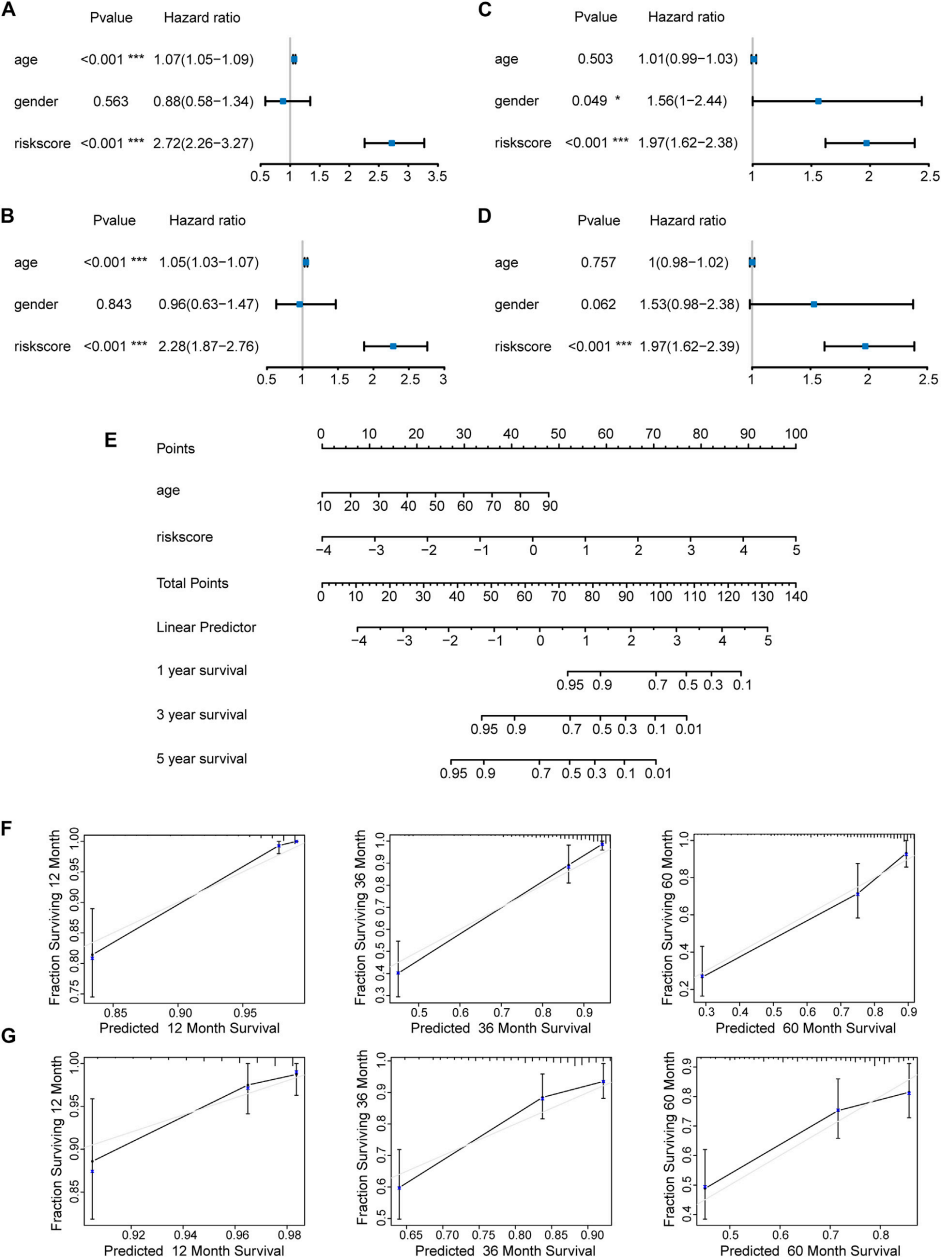
Construction and evaluation of a nomogram. Independent prognostic factors were identified by univariate and multivariate Cox regression analyses regarding overall survival in the TCGA cohort **(A,B)** and the CGGA cohort **(C,D)** (*p < 0.05; ***p < 0.001). **(E)** Construction of a nomogram based on the independent prognostic values in the TCGA cohort. The calibration curves between predicted and observed 1-year, 3-year, and 5-year outcomes of nomograms in the TCGA cohort **(F)** and the CGGA cohort **(G)**. Gray diagonal line represented ideal prediction.

We first evaluated the prognostic value of age, gender, and risk score in patients with LGG from the TCGA cohort through univariate Cox regression analysis. The result revealed that both age (HR = 1.07, 95% CI = 1.05–1.09, *p* < 0.001) and risk score (HR = 2.72, 95% CI = 2.26–3.27, *p* < 0.001) of LGG patients were significantly correlated with overall survival (Figure 7A). Moreover, multivariate Cox regression analysis showed that age (HR = 1.05, 95% CI = 1.03–1.07, *p* < 0.001) and risk score (HR = 2.28, 95% CI = 1.87–2.76, *p* < 0.001) affected overall survival as independent prognostic factors (Figure 7B). Similar results were obtained when univariate and multivariate Cox regression analyses were applied in LGG patients from the CGGA cohort. Of note, gender (HR = 1.56, 95% CI = 1–2.44, *p* = 0.049) and risk score (HR = 1.97, 95% CI = 1.62–2.38, *p* < 0.001) were correlated with overall survival, but only risk score (HR = 1.97, 95% CI = 1.62–2.39, *p* < 0.001) became an independent prognostic factor in the CGGA cohort (Figures 7C,D). The subtle difference may be attributed to the ethnicity difference.

On the basis of the seven-E3RG risk signature, we established a nomogram that could predict the prognosis of LGG in the TCGA cohort (Figure 7E). Briefly, the points of different variables were mapped to the corresponding lines, while the total points of the patients were calculated and normalized to a distribution of 0–100. By performing this, the 1-, 3-, and 5-year survival status for LGG patients could be approximately estimated based on the prognosis axis and total point axis. In addition, the calibration curves for the probability of 1-, 3-, and 5-year overall survival showed a strong consistency between the predicted value of the nomogram and the actual value in both the TCGA and CGGA cohorts (Figures 7F,G). Thus, the nomogram could serve as a favorable reference for clinical decision-making.

### Identification and Function Analysis of Risk-Related Differentially Expressed Genes

**FIGURE 8:**
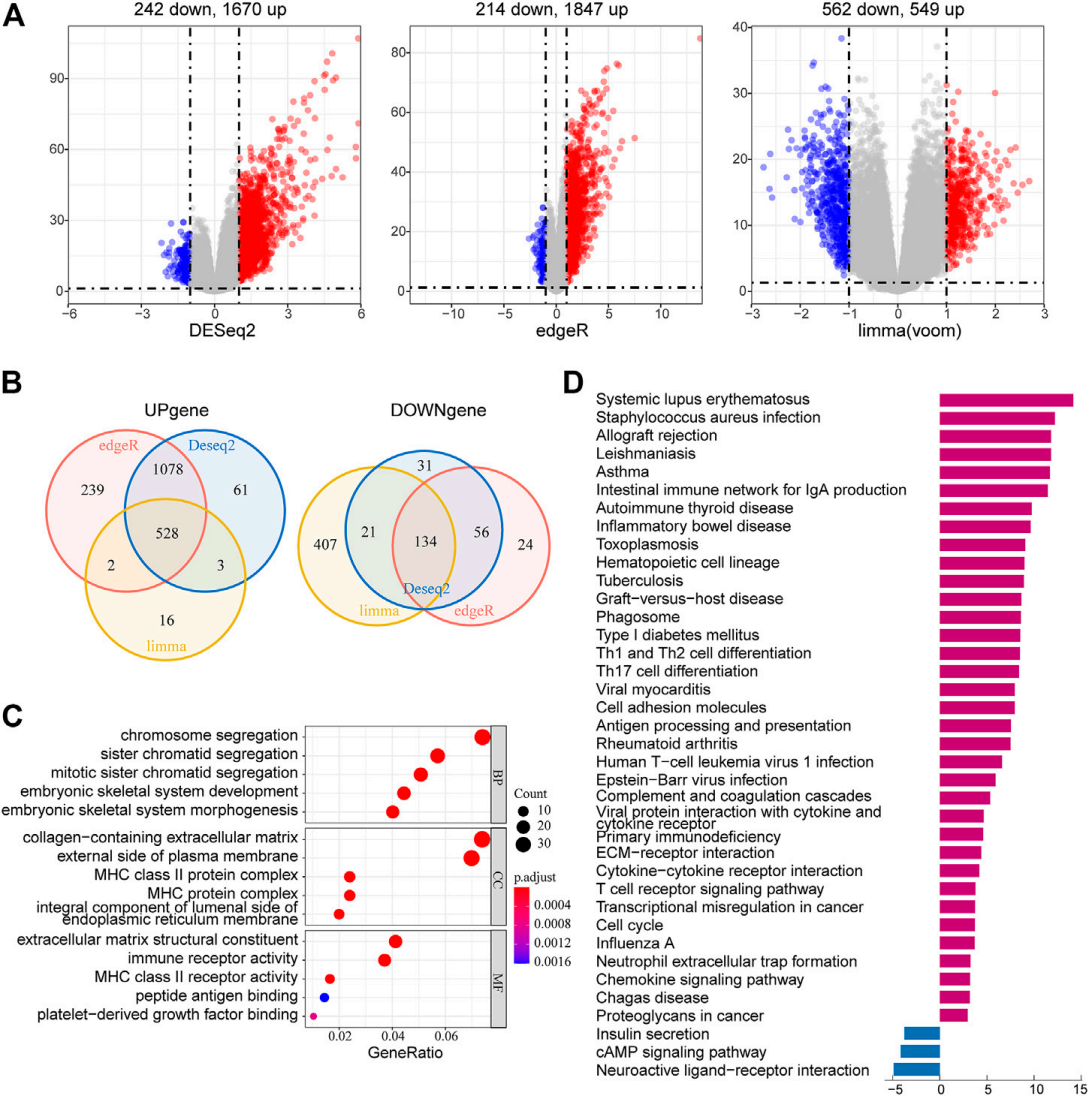
Identification and functional enrichment analyses of risk-related DEGs. **(A)** Volcano plot of risk-related DEGs between the high-risk group and low-risk group identified using edgeR, limma, and DESeq2 algorithms, with the cut-off criterion *p* < 0.05 and |log_2_FC| ≥ 1. Blue dots: significantly downregulated genes; red dots: significantly upregulated genes. **(B)** Venn diagram of the overlapping risk-related DEGs screened by the three differential expression analyses. **(C)** GO analysis of risk-related DEGs with three terms biological processes, cellular components, and molecular functions (P.adjust < 0.05). **(D)** KEGG pathways enriched in the upregulated and downregulated risk-related DEGs (P.adjust < 0.01).

To further investigate the potential biological functions and pathways of the risk signature, we screened the DEGs by three differential expression analyses between the high-risk group and the low-risk group in the TCGA LGG cohort. These results were displayed in volcano plots (Figure 8A). As shown by the Venn diagrams in Figure 8B, 528 upregulated genes and 134 downregulated genes in the high-risk group were identified and applied for GO and KEGG pathway analyses (Figure 8B). From the GO analysis, the risk-related DEGs were enriched in extracellular matrix–related functions, which suggested stronger migration and invasion potentials of tumors for LGG patients in the high-risk group, such as collagen-containing extracellular matrix and extracellular matrix structural constituent. Meanwhile, the risk-related DEG high enrichment was observed in several immune-related biological processes, such as MHC class II protein complex, MHC protein complex, MHC class II receptor activity, and immune receptor activity (Figure 8C). Moreover, the KEGG pathway enrichment analysis showed that risk-related DEGs were principally intensified in immune-related pathways and cell adhesion molecules, which reflected the malignant characteristics of LGG in the high-risk group (Figure 8D). This was also consistent with the results of GO analysis.

### GSEA Enrichment Analysis

**FIGURE 9:**
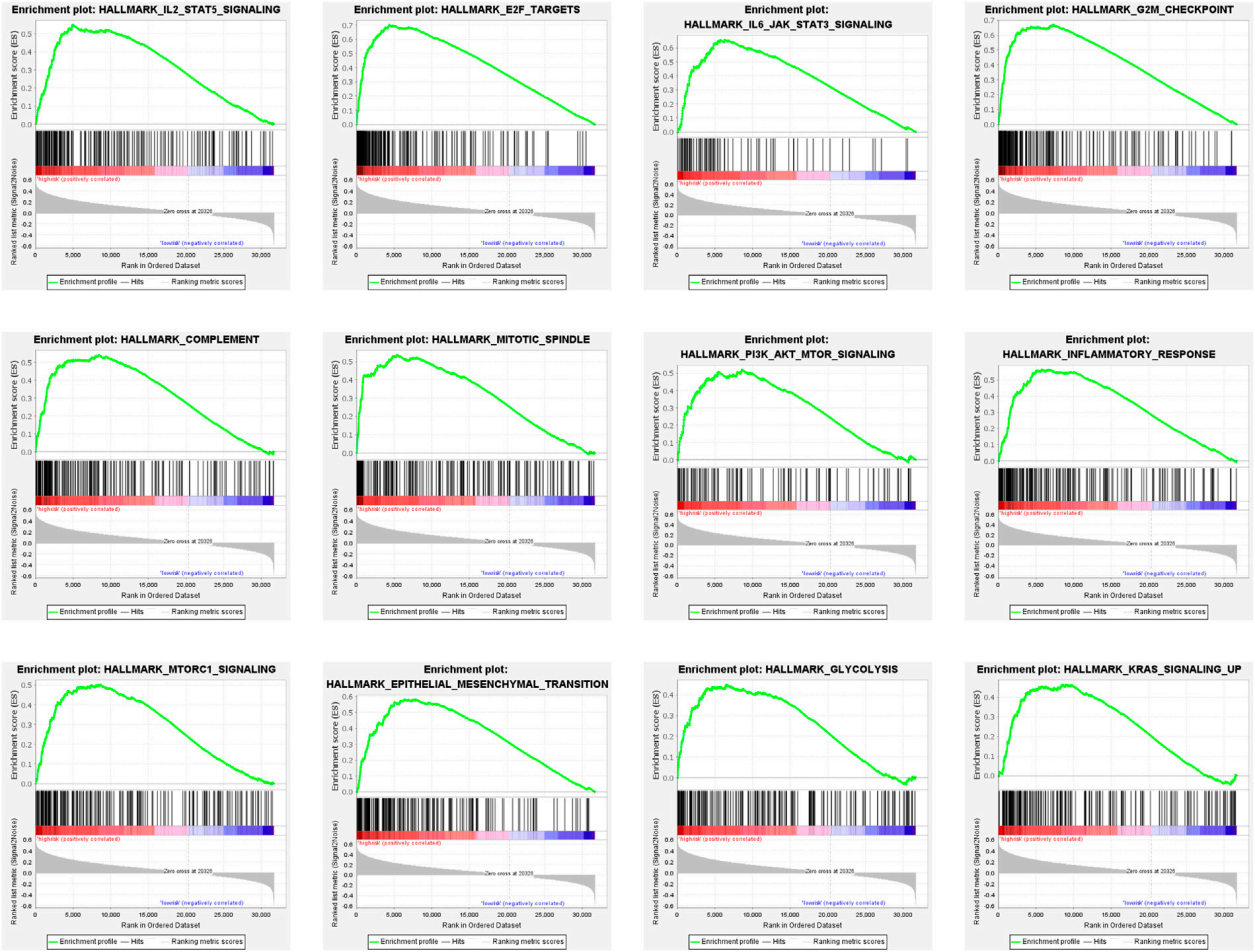
Gene set enrichment analysis (GSEA) of DEGs in the high-risk group.

To elucidate the potential functional differences between the high-risk and low-risk groups, GSEA was performed with the TCGA LGG cohort. GSEA revealed that pathways related to inflammatory response, such as complement, IL-2/STAT5 signaling, IL-6/JAK/STAT3 signaling, and inflammatory response, were enriched in the high-risk group. Pathways that promote tumorigenesis and progression were also enriched in the high-risk group, such as PI3K/AKT/MTOR signaling, mTORC1 signaling, epithelial–mesenchymal transition, glycolysis, and KRAS signaling up. The enrichment of genes related to E2F targets, G2M checkpoint, and mitotic spindle suggested the correlation of cell cycle dysregulation with the risk score (Figure 9).

### The Role of the E3-Related Gene Risk Signature in the Tumor Immune Microenvironment

**FIGURE 10:**
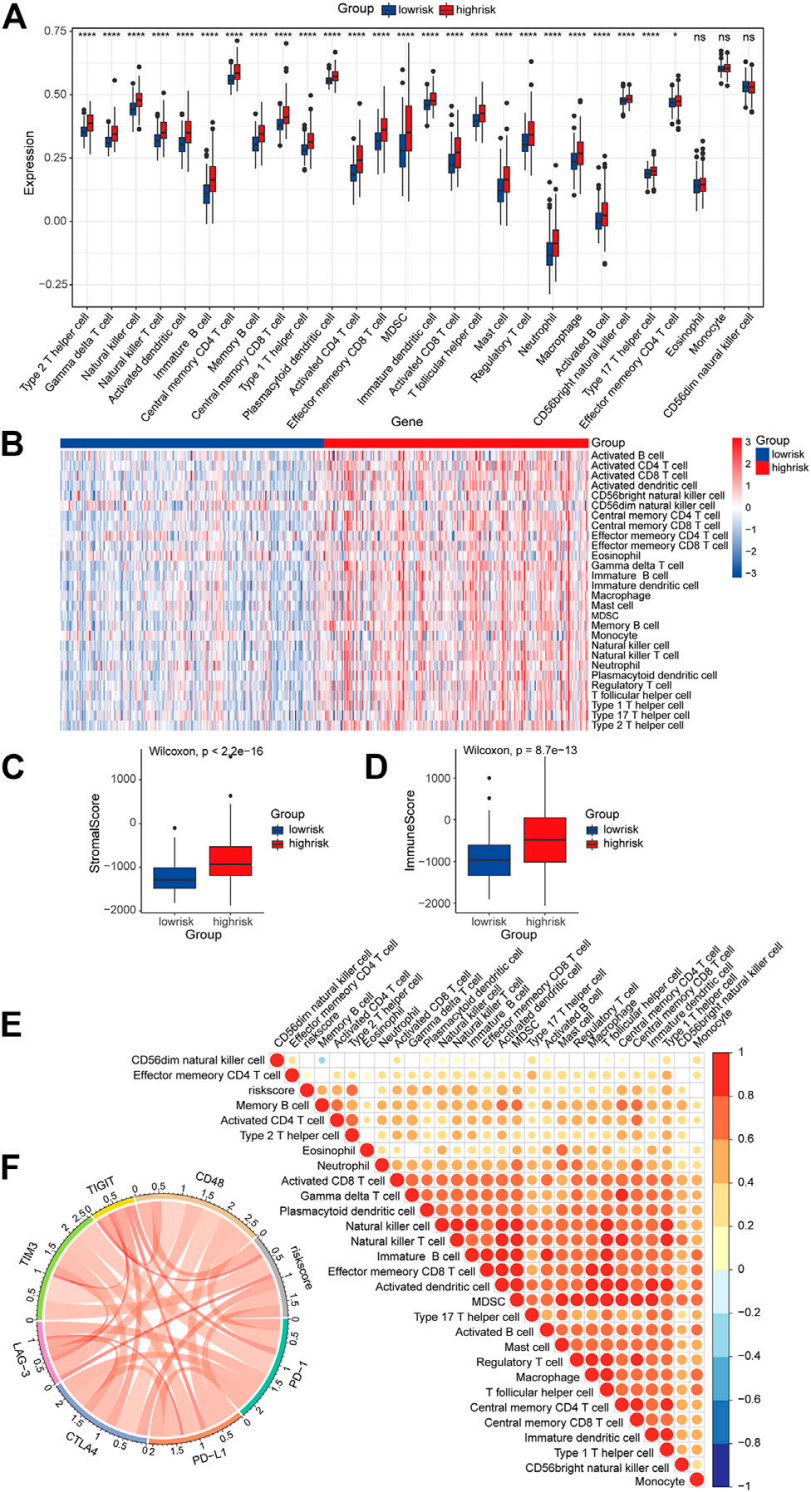
Role of the risk signature in the TIME. **(A)** ssGSEA scores of the 28 immune cells in the high-risk group and the low-risk group (*p < 0.05; ****p < 0.0001; ns, not significant). **(B)** Heatmap of the expression of the immune cell markers in the low-risk and the high-risk groups. **(C,D)** Differences in overall immune and stromal activity between the high-risk group and the low-risk group using the ESTIMATE algorithm. **(E)** Correlation between the risk signature and 28 TIME cell infiltration. **(F)** Correlation between the risk signature and immune checkpoints.

In order to further investigate the roles of the risk signature in TIME cell infiltration, we evaluated the landscape of 28 TIME-infiltrating cell types in the low-risk and high-risk groups by ssGSEA (Charoentong et al., 2017). In total, 25 TIME cell types presented significant differences in infiltration between low-risk and high-risk groups (Figure 10A). Although eosinophils, monocytes, and CD56 dim natural killer cells were not highly enriched in the high-risk group, a mild increase was still noted in eosinophils and monocytes. The expression of immune cell markers was displayed in a heatmap (Figure 10B). Next, the ESTIMATE algorithm was applied to evaluate the immune and stromal activity in the LGG tumor microenvironment. The results disclosed that the immune and stromal activities were significantly elevated in the high-risk group, which might provide evidence for the contribution of an inflammatory environment of LGG as well (Figures 10C,D). Correlation analysis was performed, and potential relations between the risk score signature and each TIME cell type are shown in Figure 10E. A significant positive correlation between the risk score and TIME cell infiltration was observed, except for eosinophils, monocytes, and CD56 dim natural killer cells. The risk score was proven to be positively correlated with the expression of immune checkpoint molecules (Figure 10F), implying the meaningful roles of risk score signature in predicting the possible response of LGG patients to clinical immunotherapy.

### Function Analysis of Substrates of E3-Related Gene Risk Signature in Low-Grade Gliomas

**FIGURE 11:**
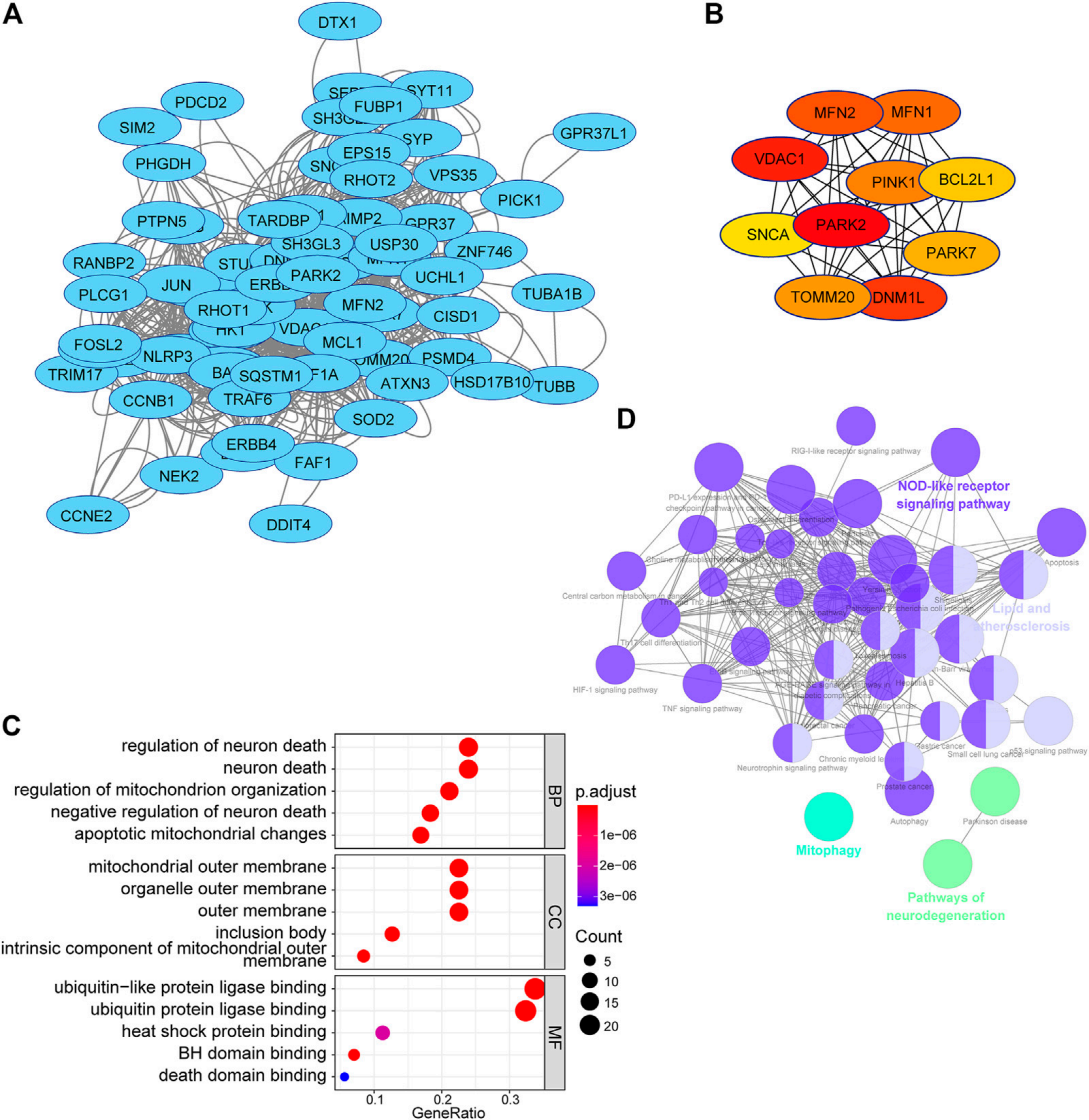
PPI network and functional analysis of substrates of the E3RG signature in LGG. **(A)** PPI network of substrates of the E3RG signature obtained with interaction scores > 0.4 based on the STRING online database and visualized using Cytoscape. **(B)** Top 10 hub genes identified using the MCC algorithm of the cytoHubba plugin in Cytoscape. **(C)** GO analysis of substrates of the E3RG signature with three terms biological processes, cellular components, and molecular functions (P.adjust < 0.05). **(D)** KEGG pathways enriched in the substrates of the E3RG signature analyzed using ClueGO plugin in Cytoscape (*p* < 0.01).

To acquire a better understanding of the potential biological function of the risk signature, the potential substrates of E3-related gene signature were searched on Ubibrowser 2.0 (http://ubibrowser.bio-it.cn/ubibrowser_v3/). The known substrates were applied for protein–protein interaction network analysis using the STRING database (https://cn.string-db.org/). A PPI network of 76 substrates of the risk signature, including 69 nodes and 772 edges, was constructed using the STRING database (Figure 11A). The top 10 hub genes with the highest linkage degrees were obtained using the MCC algorithm of the cytoHubba plugin in Cytoscape3.9.1. These genes included PARK2, VDAC1, DNM1L, MFN2, MFN1, PINK1, TOMM20, PARK7, BCL2L1, and SNCA (Figure 11B). To disclose the potential biological functions of the substrates that were involved, GO and KEGG pathway analyses were performed. GO analysis presented high enrichment in neuron death (regulation of neuron death, neuron death, negative regulation of neuron death, apoptotic mitochondrial changes, and death domain binding) and ubiquitin-related functions (ubiquitin-like protein ligase binding and ubiquitin–protein ligase binding) (Figure 11C). KEGG pathway analysis results demonstrated that the substrates were mostly correlated with neurodegeneration (pathways of neurodegeneration, Parkinson’s disease, and amyotrophic lateral sclerosis), cell death (mitophagy, apoptosis, and autophagy), and immune response (NOD-like receptor signaling pathway, PD-L1 expression, and PD-1 checkpoint pathway in cancer) (Figure 11D).

## Discussion

In the past few decades, overwhelming evidence indicates that E3 ubiquitin ligases play pivotal roles in tumorigenesis, cancer progression, and treatment responses. Since E3 ligases determine the targets of the UPS, they play an essential role in cellular functions. They take part in biological processes, including but not limited to apoptosis, cell growth, senescence, proliferation, immune system evasion, metabolism, DNA repair, inflammation, invasion, metastasis, and angiogenesis. In GBM, alterations in EGFR are commonly seen (Brennan et al., 2013). EGFR could be downregulated by PARK2 but increased by TRIM11; both are E3 ligases possessing opposite effects on EGFR (Di et al., 2013; Lin et al., 2015). The PI3K/Akt pathway is altered in approximately 90% of GBM cases (Brennan et al., 2013). The PI3K/Akt signaling could be modulated by the SCF^β−TrCP^ complex and SCF^Skp2^ complex and regulate the proliferation of primary GBM cell lines, glioma stem cells (GSCs), and established GBM cell line models (Winston et al., 1999; Li et al., 2009; Yang et al., 2009; Chan et al., 2012; Feng et al., 2014). Hence, more and more E3 ubiquitin ligases emerge as potential targets of drug designs for cancer therapies as they own better specificity for the recognition of substrates.

In this study, we first identified 38 DEGs with survival significance, in which seven DEGs showed strong prognostic performance and constituted a risk signature. The signature consists of AURKA, MAP3K1, TRIM34, PCGF2, PRKN, TLE3, and TRIM17. Patients in the high-risk group were more likely to have a worse prognosis compared with the ones in the low-risk group. Among the seven genes of the risk signature, many have been reported in glioma pathogenesis. Aurora kinase A (AURKA) has emerged as a drug target for glioblastoma for being highly involved in cell proliferation, migration, and invasion (Wang et al., 2018; Nguyen et al., 2021). MAP3K1 might promote glioma stem cell progression and be positively associated with resistance to temozolomide (TMZ) and radiotherapy (Bi et al., 2020; Wang et al., 2020). PRKN, first found to be mutated in patients with early-onset Parkinson’s disease, has also been confirmed to carry mutations and deletions in human malignancies including glioblastoma, colon cancer, and lung cancers (Veeriah et al., 2010). In addition, PRKN inhibits glioma cell growth *in vitro* and *in vivo* by downregulating the intracellular levels of β-catenin and EGFR, leading to decreased activation of both Wnt- and EGF-stimulated pathways (Lin et al., 2015). Although having not been reported in glioma-related research, TRIM34, PCGF2, TLE3, and TRIM17 have been revealed to contribute to the carcinogenesis of other cancers. TRIM34 appears to attenuate colon inflammation and tumorigenesis by sustaining barrier integrity, highlighting its role in immune responses (Lian et al., 2021). PCGF2 serves as a tumor suppressor in breast cancer, gastric cancer, and colon cancer probably for the negative regulation of Akt activation (Wang et al., 2009; Guo et al., 2010; Zhang et al., 2010). TLE3 expression is positively correlated with taxane sensitivity in patients with ovarian carcinoma but not breast cancer (Samimi et al., 2012; Bartlett et al., 2015). TRIM17 augments BRAF-targeted therapy sensitivity of melanoma cells by preventing BCL2A1 from being ubiquitinated and degraded by TRIM28 (Lionnard et al., 2019). The studies mentioned earlier once again address the potent roles of our risk genes in modulating cancer biological functions.

We screened the DEGs between high-risk and low-risk groups and performed the GO and KEGG pathway analyses. GO analysis revealed that the DEGs were enriched in extracellular matrix–related (ECM-related) functions in the high-risk group. The glioma ECM has several unique characteristics that make it distinct from the ECM of normal brain tissue. Glioma cells express components such as tenascin-C, fibronectin, and thrombospondin, which support the adhesion and migration of glioma cells. Furthermore, signals driven by the ECM also help shape tumor phenotypes (Sood et al., 2019). Our result corroborated that glioma ECM is tightly related to tumor cell proliferation and differentiation and poor prognosis. In addition, DEG high enrichment was observed in several immune-related biological processes such as MHC-related complex and immune receptor activity. The glioma TIME has diverse cell types and immune cell infiltration which consequently create a field of dynamic cytokine and chemokine communication. MHCII is expressed in different types of gliomas and is associated with increased infiltration of T cells. Both clinical and transcriptomic data have uncovered that tumoral MHCII is tightly correlated with poor prognosis and immune responses (Chih et al., 2021, 01). The KEGG pathway enrichment analysis showed that risk-related DEGs were primarily intensified in immune-related pathways and cell adhesion molecules. Accordingly, both pathways contribute to the invasion and metastasis of glioma.

GSEA results showed that pathways related to inflammatory responses were enriched in the high-risk group. Previous research indicates that about 15–20% of cancer cases suffer infection, chronic inflammation, or autoimmunity in the same tissue before solid tumor formation (Grivennikov et al., 2010). Meanwhile, the inflammatory nature of the tumor microenvironment promotes the development and survival of tumors (Mantovani et al., 2008). IL-2/STAT5 signaling is crucial for the regulation of regulatory T cells and immune tolerance (Shi et al., 2018). Similarly, the IL6-JAK-STAT3 signaling pathway may promote tumor cell proliferation, invasion, and metastasis and suppress immune response (Johnson et al., 2018). In addition, inflammatory responses and complement-related pathways could affect the tumorigenesis, immune surveillance, and immunotherapy response (Greten and Grivennikov, 2019). Our study showed the enrichment of the immune-related pathways in the high-risk group, which emphasized their significant roles in glioma prognosis. Furthermore, pathways that facilitate tumorigenesis and progression were also enriched in the high-risk group. The PI3K/AKT/mTOR pathway is widely dysregulated almost in all human cancers and is pivotal to cancer cell proliferation, survival, and therapy resistance (Cirone, 2021; Pungsrinont et al., 2021). Mutations within genes of this signaling pathway are the most common events occurring in solid malignancies including glioma (Baghery Saghchy Khorasani et al., 2021). mTORC1, one of the mTOR forms, comprises mTOR, raptor, GβL, and deptor. In particular, mTORC1 signaling is mainly involved in cell growth and metabolism (Unni and Arteaga, 2019). Furthermore, AKT/PI3K signaling could indirectly activate mTORC1 by the phosphorylation of PRAS40, a known mTORC1 inhibitor (Sancak et al., 2007; Thedieck et al., 2007; Vander Haar et al., 2007; Wang et al., 2007). Activated mTORC1 signaling may trigger recurrent reprograming that helps escape from glycolytic addiction in cancer cell lines from various solid tumor types (Pusapati et al., 2016). Recently, treatments targeting PI3K/AKT/mTOR and mTORC1 pathways have emerged as promising strategies in cancer therapeutics (Yang et al., 2019; Peng et al., 2022). Epithelial–mesenchymal transition (EMT) is a process that the majority of tumors have gone through during tumor progression. The role of EMT in tumorigenesis has been extensively investigated in different cancers including glioma (Lee et al., 2006; Phillips et al., 2006; Hugo et al., 2007; Thiery et al., 2009; Zarkoob et al., 2013). Activated EMT has also been found to be associated with the generation of cancer stem cells (Ye and Weinberg, 2015). KRAS gene polymorphisms are associated with the risk of glioma (Guan et al., 2021). Collectively, GSEA results suggested that LGG patients with high-risk scores tend to develop a faster deterioration than patients with the low-risk scores.

Recently, a growing body of studies has demonstrated how E3 ligases function in the tumor microenvironment (Do et al., 2022; Hosein et al., 2022; Iwamoto et al., 2022). The tumor microenvironment consists of different cells, including tumor cells, tumor stem cells, and stromal cells. These cells form a complex network and interact with one another to regulate tumor malignant behaviors and treatment resistance. Growth factors, chemokines, and cytokines released by immune cells are widely involved in tumor progression and therapy responses (Bindea et al., 2013). Here, we assessed the infiltration level of 28 TIME immune cells in the high- and low-risk groups to explore the roles of identified signature genes. By ssGSEA, our results showed that 25 out of 28 immune cells are more abundant in the high-risk group than in the low-risk group. In line with other studies, macrophages seem to constitute a majority of infiltration in low-grade gliomas (Rossi et al., 1988; Mieczkowski et al., 2015). These could be important findings since it has been shown that the infiltration of macrophages is highly associated with shorter overall survival in low-grade glioma (Müller et al., 2017). Our study observed the increase of most immune cells infiltrated in the high-risk group, which might be correlated with poor prognosis. The immune activity and stromal activity were remarkably elevated in the high-risk group, which once again reiterated the heterogeneity of the glioma TIME between the two groups. Determining the roles of the signature genes in TIME cell infiltration heterogeneity will be beneficial to better understand the mechanisms of the TIME antitumor immune response and developing novel immunotherapy strategies (Kim et al., 2022; Ogino et al., 2022; Tian et al., 2022).

Previous studies have identified different immune checkpoint molecules in gliomas, such as CTLA-4, TIM-3, PD-1, CD48, and LAG3 (Chouaib, 2020). Immunotherapy-targeting immune checkpoint proteins have been found to trigger an antitumor immune response (Zeng et al., 2013; Reardon et al., 2016; Boussiotis and Charest, 2018). Therefore, we evaluated the correlation between the risk score and the expression of immune checkpoint molecules. It is worth noting that the risk scores were positively correlated with the expression of immune checkpoint molecules. These results implied the involvement of the signature genes in the pathogenesis of the gliomas *via* regulating immune-related pathways. Accordingly, a preliminary function analysis for the predicted substrates of the risk signature was used. The results suggested that the substrates might regulate the pathways of cell death and immune responses. This partially explained better responses of individuals with high-risk scores to immunotherapies. Yet, these results further verified the reliability of the risk model in predicting LGG prognosis.

Here, we constructed a prognostic model based on E3RGs and a relevant nomogram in LGG. Of note, we identified a risk signature in the TCGA dataset, which consisted predominately of Caucasian and African American cases. Then, we validated the effect of the risk signature in the CGGA dataset which consisted of Chinese patients. The similar survival analysis results observed in both training and validation sets proved that our model could predict LGG prognosis in varying ethnicities. Therefore, the predictive capability of this model could be beneficial to clinical decision-making with LGG patients. Nevertheless, the construction and validation of the risk model were accomplished by retrospective analysis. Prospective clinical research needs to be rendered for further validation of this model. Moreover, the molecular mechanism of the genes in the risk model requires in-depth investigation in the future.

## Conclusion

In summary, by differential expression analyses following univariate Cox regression analysis and log-rank statistical test, E3-related DEGs with a prognostic value were identified. A seven-gene risk signature was constructed using the LASSO-Cox regression model. The risk signature achieved good performance in predicting the prognosis of LGG. Patients with high-risk scores were more likely to have a poor prognosis compared with the ones with low-risk scores. Functional analyses of the risk signature were performed. This study is expected to benefit the diagnosis and the potential therapeutics of low-grade glioma.

## Data Availability

The datasets presented in this study can be found in online repositories. The names of the repository/repositories and accession number(s) can be found in the article/Supplementary Material.

## Abbreviations

WHO, World Health Organization; LGG, low-grade glioma; GBM, glioblastoma multiforme; PTMs, posttranslational modifications; UPS, ubiquitin-proteasome system; DUBs, deubiquitinating enzymes; E3RGs, E3-related genes; PROTAC, proteolysis targeting chimeric; TCGA, The Cancer Genome Atlas; CPM, counts per million reads mapped; FPKM, Fragments Per Kilobase of transcript per Million mapped reads; DEGs, differentially expressed genes; LASSO, least absolute shrinkage and selection operator; HRs, hazard ratios; CIs, confidence intervals; ROC, receiver operating characteristic; GO, Gene Ontology; KEGG, Kyoto Encyclopedia of Genes and Genomes; BP, biological process; CC, cellular component; MF, molecular function; GSEA, gene set enrichment analysis; ssGSEA, single-sample gene set enrichment analysis; PPI, protein–protein interaction network; AUC, area under the curve; TIME, tumor immune microenvironment; GSCs, glioma stem cells; TMZ, temozolomide; MHC, major histocompatibility complex; ECM, extracellular matrix.

## References

[B1] Baghery Saghchy Khorasani A. Pourbagheri-Sigaroodi A. Pirsalehi A. Safaroghli-Azar A. Zali M. R. Bashash D. (2021). The PI3K/Akt/mTOR Signaling Pathway in Gastric Cancer; from Oncogenic Variations to the Possibilities for Pharmacologic Interventions. Eur. J. Pharmacol. 898, 173983. 10.1016/j.ejphar.2021.173983 33647255

[B2] Bartlett J. M. S. Nielsen T. O. Nielsen T. O. Gao D. Gelmon K. A. Quintayo M. A. (2015). TLE3 Is Not a Predictive Biomarker for Taxane Sensitivity in the NCIC CTG MA.21 Clinical Trial. Br. J. Cancer 113, 722–728. 10.1038/bjc.2015.271 26284338 PMC4559832

[B3] Bi C.-L. Liu J.-F. Zhang M.-Y. Lan S. Yang Z.-Y. Fang J.-S. (2020). LncRNA NEAT1 Promotes Malignant Phenotypes and TMZ Resistance in Glioblastoma Stem Cells by Regulating let-7g-5p/MAP3K1 axis. Biosci. Rep. 40, BSR20201111. 10.1042/BSR20201111 33057597 PMC7601351

[B4] Bindea G. Mlecnik B. Tosolini M. Kirilovsky A. Waldner M. Obenauf A. C. (2013). Spatiotemporal Dynamics of Intratumoral Immune Cells Reveal the Immune Landscape in Human Cancer. Immunity 39, 782–795. 10.1016/j.immuni.2013.10.003 24138885

[B5] Blanche P. Dartigues J.-F. Jacqmin-Gadda H. (2013). Estimating and Comparing Time-dependent Areas under Receiver Operating Characteristic Curves for Censored Event Times with Competing Risks. Stat. Med. 32, 5381–5397. 10.1002/sim.5958 24027076

[B6] Boussiotis V. A. Charest A. (2018). Immunotherapies for Malignant Glioma. Oncogene 37, 1121–1141. 10.1038/s41388-017-0024-z 29242608 PMC5828703

[B7] Brat D. J. Verhaak R. G. W. Aldape K. D. Yung W. K. A. Salama S. R. Cooper L. A. D. (2015). Comprehensive, Integrative Genomic Analysis of Diffuse Lower-Grade Gliomas. N. Engl. J. Med. 372, 2481–2498. 10.1056/NEJMoa1402121 26061751 PMC4530011

[B8] Brennan C. W. Verhaak R. G. McKenna A. Campos B. Noushmehr H. Salama S. R. (2013). The Somatic Genomic Landscape of Glioblastoma. Cell 155, 462–477. 10.1016/j.cell.2013.09.034 24120142 PMC3910500

[B9] Buetow L. Huang D. T. (2016). Structural Insights into the Catalysis and Regulation of E3 Ubiquitin Ligases. Nat. Rev. Mol. Cell Biol. 17, 626–642. 10.1038/nrm.2016.91 27485899 PMC6211636

[B10] Chan C.-H. Li C.-F. Yang W.-L. Gao Y. Lee S.-W. Feng Z. (2012). The Skp2-SCF E3 Ligase Regulates Akt Ubiquitination, Glycolysis, Herceptin Sensitivity, and Tumorigenesis. Cell 151, 913–914. 10.1016/j.cell.2012.10.025 30360292

[B11] Charoentong P. Finotello F. Angelova M. Mayer C. Efremova M. Rieder D. (2017). Pan-cancer Immunogenomic Analyses Reveal Genotype-Immunophenotype Relationships and Predictors of Response to Checkpoint Blockade. Cell Rep. 18, 248–262. 10.1016/j.celrep.2016.12.019 28052254

[B12] Chih Y. Sahm K. Sadik A. Bunse T. Trautwein N. Pusch S. (2021). KS01.3.A Tumoral MHC Class II Expression in Gliomas Drives T Cell Exhaustion. Neuro-Oncology 23, ii3. ii3–ii3. 10.1093/neuonc/noab180.007

[B13] S. Chouaib, (Editor) (2020). Autophagy in Immune Response: Impact on Cancer Immunotherapy (London ; San Diego, CA: Academic Press).

[B14] Cirone M. (2021). Cancer Cells Dysregulate PI3K/AKT/mTOR Pathway Activation to Ensure Their Survival and Proliferation: Mimicking Them Is a Smart Strategy of Gammaherpesviruses. Crit. Rev. Biochem. Mol. Biol. 56, 500–509. 10.1080/10409238.2021.1934811 34130564

[B15] Claus E. B. Walsh K. M. Wiencke J. K. Molinaro A. M. Wiemels J. L. Schildkraut J. M. (2015). Survival and Low-Grade Glioma: the Emergence of Genetic Information. Foc 38, E6. 10.3171/2014.10.FOCUS12367 PMC4361022 25552286

[B16] Deng L. Meng T. Chen L. Wei W. Wang P. (2020). The Role of Ubiquitination in Tumorigenesis and Targeted Drug Discovery. Sig Transduct. Target Ther. 5, 11. 10.1038/s41392-020-0107-0 PMC7048745 32296023

[B17] Di K. Linskey M. E. Bota D. A. (2013). TRIM11 Is Overexpressed in High-Grade Gliomas and Promotes Proliferation, Invasion, Migration and Glial Tumor Growth. Oncogene 32, 5038–5047. 10.1038/onc.2012.531 23178488 PMC3766389

[B18] Do T. T. Yeh C.-C. Wu G.-W. Hsu C.-C. Chang H.-C. Chen H.-C. (2022). TRIM37 Promotes Pancreatic Cancer Progression through Modulation of Cell Growth, Migration, Invasion, and Tumor Immune Microenvironment. Ijms 23, 1176. 10.3390/ijms23031176 35163097 PMC8835669

[B19] Feng H. Lopez G. Y. Kim C. K. Alvarez A. Duncan C. G. Nishikawa R. (2014). EGFR Phosphorylation of DCBLD2 Recruits TRAF6 and Stimulates AKT-Promoted Tumorigenesis. J. Clin. Invest. 124, 3741–3756. 10.1172/JCI73093 25061874 PMC4151226

[B20] Friedman J. Hastie T. Tibshirani R. (2010). Regularization Paths for Generalized Linear Models via Coordinate Descent. J. Stat. Softw. 33, 1–22. 10.18637/jss.v033.i01 20808728 PMC2929880

[B21] Gittleman H. Sloan A. E. Barnholtz-Sloan J. S. (2020). An Independently Validated Survival Nomogram for Lower-Grade Glioma. Neuro Oncol. 22, 665–674. 10.1093/neuonc/noz191 31621885 PMC7229246

[B22] Greten F. R. Grivennikov S. I. (2019). Inflammation and Cancer: Triggers, Mechanisms, and Consequences. Immunity 51, 27–41. 10.1016/j.immuni.2019.06.025 31315034 PMC6831096

[B23] Grivennikov S. I. Greten F. R. Karin M. (2010). Immunity, Inflammation, and Cancer. Cell 140, 883–899. 10.1016/j.cell.2010.01.025 20303878 PMC2866629

[B24] Gu Z. Gu L. Eils R. Schlesner M. Brors B. (2014). Circlize Implements and Enhances Circular Visualization in R. Bioinformatics 30, 2811–2812. 10.1093/bioinformatics/btu393 24930139

[B25] Guan Q. Yuan L. Lin A. Lin H. Huang X. Ruan J. (2021). KRAS Gene Polymorphisms Are Associated with the Risk of Glioma: a Two-Center Case-Control Study. Transl. Pediatr. 10, 579–586. 10.21037/tp-20-359 33850816 PMC8039792

[B26] Guo B.-H. Zhang X. Zhang H.-Z. Lin H.-L. Feng Y. Shao J.-Y. (2010). Low Expression of Mel-18 Predicts Poor Prognosis in Patients with Breast Cancer. Ann. Oncol. 21, 2361–2369. 10.1093/annonc/mdq241 20444850

[B27] Haar E. V. Lee S.-I. Bandhakavi S. Griffin T. J. Kim D.-H. (2007). Insulin Signalling to mTOR Mediated by the Akt/PKB Substrate PRAS40. Nat. Cell Biol. 9, 316–323. 10.1038/ncb1547 17277771

[B28] Hänzelmann S. Castelo R. Guinney J. (2013). GSVA: Gene Set Variation Analysis for Microarray and RNA-Seq Data. BMC Bioinforma. 14, 7. 10.1186/1471-2105-14-7 PMC3618321 23323831

[B29] Hosein A. N. Dangol G. Okumura T. Roszik J. Rajapakshe K. Siemann M. (2022). Loss of Rnf43 Accelerates Kras-Mediated Neoplasia and Remodels the Tumor Immune Microenvironment in Pancreatic Adenocarcinoma. Gastroenterology 162, 1303–1318. e18. 10.1053/j.gastro.2021.12.273 34973294 PMC8934289

[B30] Hugo H. Ackland M. L. Blick T. Lawrence M. G. Clements J. A. Williams E. D. (2007). Epithelial-mesenchymal and Mesenchymal-Epithelial Transitions in Carcinoma Progression. J. Cell. Physiol. 213, 374–383. 10.1002/jcp.21223 17680632

[B31] Iwamoto A. Tsukamoto H. Nakayama H. Oshiumi H. (2022). E3 Ubiquitin Ligase Riplet Is Expressed in T Cells and Suppresses T Cell-Mediated Antitumor Immune Responses. J. I. 208, 2067–2076. 10.4049/jimmunol.2100096 35365564

[B32] Johnson D. E. O'Keefe R. A. Grandis J. R. (2018). Targeting the IL-6/JAK/STAT3 Signalling axis in Cancer. Nat. Rev. Clin. Oncol. 15, 234–248. 10.1038/nrclinonc.2018.8 29405201 PMC5858971

[B33] Kim A.-R. Choi S. J. Park J. Kwon M. Chowdhury T. Yu H. J. (2022). Spatial Immune Heterogeneity of Hypoxia-Induced Exhausted Features in High-Grade Glioma. Oncoimmunology 11, 2026019. 10.1080/2162402X.2022.2026019 35036078 PMC8757477

[B34] Lee J.-H. Liu R. Li J. Zhang C. Wang Y. Cai Q. (2017). Stabilization of Phosphofructokinase 1 Platelet Isoform by AKT Promotes Tumorigenesis. Nat. Commun. 8, 949. 10.1038/s41467-017-00906-9 29038421 PMC5643558

[B35] Lee T. K. Poon R. T. P. Yuen A. P. Ling M. T. Kwok W. K. Wang X. H. (2006). Twist Overexpression Correlates with Hepatocellular Carcinoma Metastasis through Induction of Epithelial-Mesenchymal Transition. Clin. Cancer Res. 12, 5369–5376. 10.1158/1078-0432.CCR-05-2722 17000670

[B36] Li X. Liu J. Gao T. (2009). β-TrCP-Mediated Ubiquitination and Degradation of PHLPP1 Are Negatively Regulated by Akt. Mol. Cell Biol. 29, 6192–6205. 10.1128/MCB.00681-09 19797085 PMC2786696

[B37] Lian Q. Yan S. Yin Q. Yan C. Zheng W. Gu W. (2021). TRIM34 Attenuates Colon Inflammation and Tumorigenesis by Sustaining Barrier Integrity. Cell Mol. Immunol. 18, 350–362. 10.1038/s41423-020-0366-2 32094504 PMC8027410

[B38] Lin D.-C. Xu L. Chen Y. Yan H. Hazawa M. Doan N. (2015). Genomic and Functional Analysis of the E3 Ligase PARK2 in Glioma. Cancer Res. 75, 1815–1827. 10.1158/0008-5472.CAN-14-1433 25877876 PMC4417379

[B39] Lionnard L. Duc P. Brennan M. S. Kueh A. J. Pal M. Guardia F. (2019). TRIM17 and TRIM28 Antagonistically Regulate the Ubiquitination and Anti-apoptotic Activity of BCL2A1. Cell Death Differ. 26, 902–917. 10.1038/s41418-018-0169-5 30042493 PMC6461866

[B40] Liu Z. Guo C. Dang Q. Wang L. Liu L. Weng S. (2022a). Integrative Analysis from Multi-Center Studies Identities a Consensus Machine Learning-Derived lncRNA Signature for Stage II/III Colorectal Cancer. EBioMedicine 75, 103750. 10.1016/j.ebiom.2021.103750 34922323 PMC8686027

[B41] Liu Z. Liu L. Weng S. Guo C. Dang Q. Xu H. (2022b). Machine Learning-Based Integration Develops an Immune-Derived lncRNA Signature for Improving Outcomes in Colorectal Cancer. Nat. Commun. 13, 816. 10.1038/s41467-022-28421-6 35145098 PMC8831564

[B42] Liu Z. Weng S. Xu H. Wang L. Liu L. Zhang Y. (2021). Computational Recognition and Clinical Verification of TGF-β-Derived miRNA Signature with Potential Implications in Prognosis and Immunotherapy of Intrahepatic Cholangiocarcinoma. Front. Oncol. 11, 757919. 10.3389/fonc.2021.757919 34760703 PMC8573406

[B43] Liu Z. Xu H. Ge X. Weng S. Dang Q. Han X. (2022c). Gene Expression Profile Reveals a Prognostic Signature of Non-MSI-H/pMMR Colorectal Cancer. Front. Cell Dev. Biol. 10, 790214. 10.3389/fcell.2022.790214 35252170 PMC8891566

[B44] Lombardi G. Barresi V. Castellano A. Tabouret E. Pasqualetti F. Salvalaggio A. (2020). Clinical Management of Diffuse Low-Grade Gliomas. Cancers 12, 3008. 10.3390/cancers12103008 PMC7603014 33081358

[B45] Love M. I. Huber W. Anders S. (2014). Moderated Estimation of Fold Change and Dispersion for RNA-Seq Data with DESeq2. Genome Biol. 15, 550. 10.1186/s13059-014-0550-8 25516281 PMC4302049

[B46] Mantovani A. Allavena P. Sica A. Balkwill F. (2008). Cancer-related Inflammation. Nature 454, 436–444. 10.1038/nature07205 18650914

[B47] Mieczkowski J. Kocyk M. Nauman P. Gabrusiewicz K. Sielska M. Przanowski P. (2015). Down-regulation of IKKβ Expression in Glioma-Infiltrating Microglia/macrophages Is Associated with Defective Inflammatory/immune Gene Responses in Glioblastoma. Oncotarget 6, 33077–33090. 10.18632/oncotarget.5310 26427514 PMC4741750

[B48] Müller S. Kohanbash G. Liu S. J. Alvarado B. Carrera D. Bhaduri A. (2017). Single-cell Profiling of Human Gliomas Reveals Macrophage Ontogeny as a Basis for Regional Differences in Macrophage Activation in the Tumor Microenvironment. Genome Biol. 18, 234. 10.1186/s13059-017-1362-4 29262845 PMC5738907

[B49] Nguyen T. T. T. Shang E. Shu C. Kim S. Mela A. Humala N. (2021). Aurora Kinase A Inhibition Reverses the Warburg Effect and Elicits Unique Metabolic Vulnerabilities in Glioblastoma. Nat. Commun. 12, 5203. 10.1038/s41467-021-25501-x 34471141 PMC8410792

[B50] Ogino H. Taylor J. W. Nejo T. Gibson D. Watchmaker P. B. Okada K. (2022). Randomized Trial of Neoadjuvant Vaccination with Tumor-Cell Lysate Induces T Cell Response in Low-Grade Gliomas. J. Clin. Invest. 132, e151239. 10.1172/JCI151239 34882581 PMC8803342

[B51] Peng Y. Wang Y. Zhou C. Mei W. Zeng C. (2022). PI3K/Akt/mTOR Pathway and its Role in Cancer Therapeutics: Are We Making Headway? Front. Oncol. 12, 819128. 10.3389/fonc.2022.819128 35402264 PMC8987494

[B52] Phillips H. S. Kharbanda S. Chen R. Forrest W. F. Soriano R. H. Wu T. D. (2006). Molecular Subclasses of High-Grade Glioma Predict Prognosis, Delineate a Pattern of Disease Progression, and Resemble Stages in Neurogenesis. Cancer Cell 9, 157–173. 10.1016/j.ccr.2006.02.019 16530701

[B53] Popovic D. Vucic D. Dikic I. (2014). Ubiquitination in Disease Pathogenesis and Treatment. Nat. Med. 20, 1242–1253. 10.1038/nm.3739 25375928

[B54] Pungsrinont T. Kallenbach J. Baniahmad A. (2021). Role of PI3K-AKT-mTOR Pathway as a Pro-survival Signaling and Resistance-Mediating Mechanism to Therapy of Prostate Cancer. Ijms 22, 11088. 10.3390/ijms222011088 34681745 PMC8538152

[B55] Pusapati R. V. Daemen A. Wilson C. Sandoval W. Gao M. Haley B. (2016). mTORC1-Dependent Metabolic Reprogramming Underlies Escape from Glycolysis Addiction in Cancer Cells. Cancer Cell 29, 548–562. 10.1016/j.ccell.2016.02.018 27052953

[B56] Qi S.-M. Dong J. Xu Z.-Y. Cheng X.-D. Zhang W.-D. Qin J.-J. (2021). PROTAC: An Effective Targeted Protein Degradation Strategy for Cancer Therapy. Front. Pharmacol. 12, 692574. 10.3389/fphar.2021.692574 34025443 PMC8138175

[B57] Que T. Zheng H. Zeng Y. Liu X. Qi G. La Q. (2022). Correction to: HMGA1 Stimulates MYH9-dependent Ubiquitination of GSK-3β via PI3K/Akt/c-Jun Signaling to Promote Malignant Progression and Chemoresistance in Gliomas. Cell Death Dis. 13, 164. 10.1038/s41419-022-04547-9 35190524 PMC8861152

[B58] Rape M. (2018). Ubiquitylation at the Crossroads of Development and Disease. Nat. Rev. Mol. Cell Biol. 19, 59–70. 10.1038/nrm.2017.83 28928488

[B59] Reardon D. A. Gokhale P. C. Klein S. R. Ligon K. L. Rodig S. J. Ramkissoon S. H. (2016). Glioblastoma Eradication Following Immune Checkpoint Blockade in an Orthotopic, Immunocompetent Model. Cancer Immunol. Res. 4, 124–135. 10.1158/2326-6066.CIR-15-0151 26546453

[B60] Reyes-Turcu F. E. Ventii K. H. Wilkinson K. D. (2009). Regulation and Cellular Roles of Ubiquitin-specific Deubiquitinating Enzymes. Annu. Rev. Biochem. 78, 363–397. 10.1146/annurev.biochem.78.082307.091526 19489724 PMC2734102

[B61] Rimkus T. K. Arrigo A. B. Zhu D. Carpenter R. L. Sirkisoon S. Doheny D. (2022). NEDD4 Degrades TUSC2 to Promote Glioblastoma Progression. Cancer Lett. 531, 124–135. 10.1016/j.canlet.2022.01.029 35167936 PMC8920049

[B62] Ritchie M. E. Phipson B. Wu D. Hu Y. Law C. W. Shi W. (2015). Limma Powers Differential Expression Analyses for RNA-Sequencing and Microarray Studies. Nucleic Acids Res. 43, e47. 10.1093/nar/gkv007 25605792 PMC4402510

[B63] Robinson M. D. McCarthy D. J. Smyth G. K. (2010). edgeR: a Bioconductor Package for Differential Expression Analysis of Digital Gene Expression Data. Bioinformatics 26, 139–140. 10.1093/bioinformatics/btp616 19910308 PMC2796818

[B64] Rossi M. L. Cruz-Sanchez F. Hughes J. T. Esiri M. M. Coakham H. B. Moss T. H. (1988). Mononuclear Cell Infiltrate and HLA-DR Expression in Low Grade Astrocytomas. Acta Neuropathol. 76, 281–286. 10.1007/BF00687776 3213431

[B65] Samimi G. Ring B. Z. Ross D. T. Seitz R. S. Sutherland R. L. O'Brien P. M. (2012). TLE3 Expression Is Associated with Sensitivity to Taxane Treatment in Ovarian Carcinoma. Cancer Epidemiol. Biomarkers Prev. 21, 273–279. 10.1158/1055-9965.EPI-11-0917 22194527

[B66] Sancak Y. Thoreen C. C. Peterson T. R. Lindquist R. A. Kang S. A. Spooner E. (2007). PRAS40 Is an Insulin-Regulated Inhibitor of the mTORC1 Protein Kinase. Mol. Cell 25, 903–915. 10.1016/j.molcel.2007.03.003 17386266

[B67] Schulman B. A. Wade Harper J. (2009). Ubiquitin-like Protein Activation by E1 Enzymes: the Apex for Downstream Signalling Pathways. Nat. Rev. Mol. Cell Biol. 10, 319–331. 10.1038/nrm2673 19352404 PMC2712597

[B68] Seeler J.-S. Dejean A. (2017). SUMO and the Robustness of Cancer. Nat. Rev. Cancer 17, 184–197. 10.1038/nrc.2016.143 28134258

[B69] Shannon P. Markiel A. Ozier O. Baliga N. S. Wang J. T. Ramage D. (2003). Cytoscape: a Software Environment for Integrated Models of Biomolecular Interaction Networks. Genome Res. 13, 2498–2504. 10.1101/gr.1239303 14597658 PMC403769

[B70] Shi H. Liu C. Tan H. Li Y. Nguyen T.-L. M. Dhungana Y. (2018). Hippo Kinases Mst1 and Mst2 Sense and Amplify IL-2R-STAT5 Signaling in Regulatory T Cells to Establish Stable Regulatory Activity. Immunity 49, 899–914. e6. 10.1016/j.immuni.2018.10.010 30413360 PMC6249059

[B71] Sood D. Tang-Schomer M. Pouli D. Mizzoni C. Raia N. Tai A. (2019). 3D Extracellular Matrix Microenvironment in Bioengineered Tissue Models of Primary Pediatric and Adult Brain Tumors. Nat. Commun. 10, 4529. 10.1038/s41467-019-12420-1 31586101 PMC6778192

[B72] Stewart M. D. Ritterhoff T. Klevit R. E. Brzovic P. S. (2016). E2 Enzymes: More Than Just Middle Men. Cell Res. 26, 423–440. 10.1038/cr.2016.35 27002219 PMC4822130

[B73] Stieren E. S. El Ayadi A. Xiao Y. Siller E. Landsverk M. L. Oberhauser A. F. (2011). Ubiquilin-1 Is a Molecular Chaperone for the Amyloid Precursor Protein. J. Biol. Chem. 286, 35689–35698. 10.1074/jbc.M111.243147 21852239 PMC3195644

[B74] Szklarczyk D. Gable A. L. Lyon D. Junge A. Wyder S. Huerta-Cepas J. (2019). STRING V11: Protein-Protein Association Networks with Increased Coverage, Supporting Functional Discovery in Genome-wide Experimental Datasets. Nucleic Acids Res. 47, D607–D613. 10.1093/nar/gky1131 30476243 PMC6323986

[B75] Thedieck K. Polak P. Kim M. L. Molle K. D. Cohen A. Jenö P. (2007). PRAS40 and PRR5-like Protein Are New mTOR Interactors that Regulate Apoptosis. PLoS One 2, e1217. 10.1371/journal.pone.0001217 18030348 PMC2075366

[B76] Thiery J. P. Acloque H. Huang R. Y. J. Nieto M. A. (2009). Epithelial-mesenchymal Transitions in Development and Disease. Cell 139, 871–890. 10.1016/j.cell.2009.11.007 19945376

[B77] Tian T. Liang R. Erel-Akbaba G. Saad L. Obeid P. J. Gao J. (2022). Immune Checkpoint Inhibition in GBM Primed with Radiation by Engineered Extracellular Vesicles. ACS Nano 16, 1940–1953. 10.1021/acsnano.1c05505 35099172 PMC9020451

[B78] Unni N. Arteaga C. L. (2019). Is Dual mTORC1 and mTORC2 Therapeutic Blockade Clinically Feasible in Cancer? JAMA Oncol. 5, 1564–1565. 10.1001/jamaoncol.2019.2525 31465107

[B79] Veeriah S. Taylor B. S. Meng S. Fang F. Yilmaz E. Vivanco I. (2010). Somatic Mutations of the Parkinson's Disease-Associated Gene PARK2 in Glioblastoma and Other Human Malignancies. Nat. Genet. 42, 77–82. 10.1038/ng.491 19946270 PMC4002225

[B80] Wang J. Zuo J. Wahafu A. Wang M. d. Li R. c. Xie W. f. (2020). Combined Elevation of TRIB2 and MAP3K1 Indicates Poor Prognosis and Chemoresistance to Temozolomide in Glioblastoma. CNS Neurosci. Ther. 26, 297–308. 10.1111/cns.13197 31318172 PMC7053231

[B81] Wang L. Harris T. E. Roth R. A. Lawrence J. C. (2007). PRAS40 Regulates mTORC1 Kinase Activity by Functioning as a Direct Inhibitor of Substrate Binding. J. Biol. Chem. 282, 20036–20044. 10.1074/jbc.M702376200 17510057

[B82] Wang R. Zhang S. Chen X. Li N. Li J. Jia R. (2018). EIF4A3-induced Circular RNA MMP9 (circMMP9) Acts as a Sponge of miR-124 and Promotes Glioblastoma Multiforme Cell Tumorigenesis. Mol. Cancer 17, 166. 10.1186/s12943-018-0911-0 30470262 PMC6260852

[B83] Wang W. Yuasa T. Tsuchiya N. Ma Z. Maita S. Narita S. (2009). The Novel Tumor-Suppressor Mel-18 in Prostate Cancer: its Functional Polymorphism, Expression and Clinical Significance. Int. J. Cancer 125, 2836–2843. 10.1002/ijc.24721 19585577

[B84] Warfel N. A. Niederst M. Stevens M. W. Brennan P. M. Frame M. C. Newton A. C. (2011). Mislocalization of the E3 Ligase, β-Transducin Repeat-Containing Protein 1 (β-TrCP1), in Glioblastoma Uncouples Negative Feedback between the Pleckstrin Homology Domain Leucine-Rich Repeat Protein Phosphatase 1 (PHLPP1) and Akt. J. Biol. Chem. 286, 19777–19788. 10.1074/jbc.M111.237081 21454620 PMC3103356

[B85] Wesseling P. Capper D. (2018). WHO 2016 Classification of Gliomas. Neuropathol. Appl. Neurobiol. 44, 139–150. 10.1111/nan.12432 28815663

[B86] Winston J. T. Strack P. Beer-Romero P. Chu C. Y. Elledge S. J. Harper J. W. (1999). The SCFbeta -TRCP-Ubiquitin Ligase Complex Associates Specifically with Phosphorylated Destruction Motifs in Ikappa Balpha and Beta -catenin and Stimulates Ikappa Balpha Ubiquitination *In Vitro* . Genes & Dev. 13, 270–283. 10.1101/gad.13.3.270 9990852 PMC316433

[B87] Xia L. Fang C. Chen G. Sun C. (2018). Relationship between the Extent of Resection and the Survival of Patients with Low-Grade Gliomas: a Systematic Review and Meta-Analysis. BMC Cancer 18, 48. 10.1186/s12885-017-3909-x 29306321 PMC5756328

[B88] Yang J. Nie J. Ma X. Wei Y. Peng Y. Wei X. (2019). Targeting PI3K in Cancer: Mechanisms and Advances in Clinical Trials. Mol. Cancer 18, 26. 10.1186/s12943-019-0954-x 30782187 PMC6379961

[B89] Yang W.-L. Wang J. Chan C.-H. Lee S.-W. Campos A. D. Lamothe B. (2009). The E3 Ligase TRAF6 Regulates Akt Ubiquitination and Activation. Science 325, 1134–1138. 10.1126/science.1175065 19713527 PMC3008763

[B90] Ye X. Weinberg R. A. (2015). Epithelial-Mesenchymal Plasticity: A Central Regulator of Cancer Progression. Trends Cell Biol. 25, 675–686. 10.1016/j.tcb.2015.07.012 26437589 PMC4628843

[B91] Yoshihara K. Shahmoradgoli M. Martínez E. Vegesna R. Kim H. Torres-Garcia W. (2013). Inferring Tumour Purity and Stromal and Immune Cell Admixture from Expression Data. Nat. Commun. 4, 2612. 10.1038/ncomms3612 24113773 PMC3826632

[B92] Yu G. Wang L.-G. Han Y. He Q.-Y. (2012). clusterProfiler: an R Package for Comparing Biological Themes Among Gene Clusters. OMICS A J. Integr. Biol. 16, 284–287. 10.1089/omi.2011.0118 PMC3339379 22455463

[B93] Zangiabadi S. Abdul-Sater A. A. (2022). Regulation of the NLRP3 Inflammasome by Posttranslational Modifications. J. I. 208, 286–292. 10.4049/jimmunol.2100734 35017218

[B94] Zarkoob H. Taube J. H. Singh S. K. Mani S. A. Kohandel M. (2013). Investigating the Link between Molecular Subtypes of Glioblastoma, Epithelial-Mesenchymal Transition, and CD133 Cell Surface Protein. PLoS One 8, e64169. 10.1371/journal.pone.0064169 23734191 PMC3667082

[B95] Zeng J. See A. P. Phallen J. Jackson C. M. Belcaid Z. Ruzevick J. (2013). Anti-PD-1 Blockade and Stereotactic Radiation Produce Long-Term Survival in Mice with Intracranial Gliomas. Int. J. Radiat. Oncology*Biology*Physics 86, 343–349. 10.1016/j.ijrobp.2012.12.025 PMC3963403 23462419

[B96] Zhang X.-W. Sheng Y.-P. Li Q. Qin W. Lu Y.-W. Cheng Y.-F. (2010). BMI1 and Mel-18 Oppositely Regulate Carcinogenesis and Progression of Gastric Cancer. Mol. Cancer 9, 40. 10.1186/1476-4598-9-40 20170541 PMC2842237

[B97] Zhao B. Burgess K. (2019). PROTACs Suppression of CDK4/6, Crucial Kinases for Cell Cycle Regulation in Cancer. Chem. Commun. 55, 2704–2707. 10.1039/c9cc00163h 30758029

[B98] Zheng N. Shabek N. (2017). Ubiquitin Ligases: Structure, Function, and Regulation. Annu. Rev. Biochem. 86, 129–157. 10.1146/annurev-biochem-060815-014922 28375744

